# Classification of finger movements through optimal EEG channel and feature selection

**DOI:** 10.3389/fnhum.2025.1633910

**Published:** 2025-07-16

**Authors:** Murside Degirmenci, Yilmaz Kemal Yuce, Matjaž Perc, Yalcin Isler

**Affiliations:** ^1^Kutahya Vocational School, Kutahya Health Sciences University, Kutahya, Türkiye; ^2^Department of Computer Engineering, Alanya Alaaddin Keykubat University, Antalya, Türkiye; ^3^Faculty of Natural Sciences and Mathematics, University of Maribor, Maribor, Slovenia; ^4^Community Healthcare Center Dr. Adolf Drolc Maribor, Maribor, Slovenia; ^5^Department of Physics, Kyung Hee University, Seoul, Republic of Korea; ^6^Complexity Science Hub, Vienna, Austria; ^7^University College, Korea University, Seoul, Republic of Korea; ^8^Department of Electrical and Electronics Engineering, Alanya Alaaddin Keykubat University, Antalya, Türkiye

**Keywords:** brain-computer interfaces (BCIs), electroencephalogram (EEG), statistical-significance based feature selection, machine learning, finger movement classification

## Abstract

**Introduction:**

Electrencephalography (EEG)-based brain-computer interfaces (BCIs) have become popular as EEG is accepted as the simplest and non-invasive neuroimaging modality to record the brain's electrical activity. In the current BCI research context, apart from predicting extremity movements, recent BCI studies have been interested in accurately predicting finger movements of the same hand using different pattern recognition methods over EEG data collected based on motor imagery (MI), through which a mental image of the desired action is generated when a person ideally simulates or imagines carrying out a certain motor task. Although several pattern recognition methods have already been recommended in literature, majority of the studies focusing on classifying five finger movements, were based on study designs that neglected or excluded the idle state of brain (i.e., no mental task state) during which brain does not carry out any MI task. This study design may result in an increasing number of false positives and a significant decrease in the prediction rates and classification performance. Moreover, recent studies have focused on improving prediction performance using complex feature extraction and machine learning algorithms while ignoring comprehensive EEG channels and feature investigation in the prediction of finger movements from EEGs. Therefore, the objectives of this study are threefold: (i) to develop a more viable and practical system to predict the movements of five fingers and the no mental task (NoMT) state from EEG signals (ii) to analyze the effects of the statistical-significance based feature selection method over four different feature domains (nonlinear domain, time-domain, frequency-domain and time-frequency domain) and their combinations, and (iii) to test these feature sets with different and prominent classifiers.

**Methods:**

In this study, our major goal is not to explore the best machine algorithm performance, but to investigate the best EEG channels and features that can be used in the classification of finger movements. Hence, the comprehensive analysis of the effectiveness of EEG channels and features is performed utilizing a statistically significant feature distribution over 19 EEG channels for each feature set independently. A bulky dataset of electroencephalographic MI for EEG-based BCIs is used in this study. A total of 1102 EEG features supplied from different feature domains have been investigated. Subsequently, these features were tested with eight well-known classifiers, comprising Decision tree, Discriminant analysis, Naive Bayes, Support vector machine, k-nearest neighbor, Ensemble learning, Neural networks, and Kernel approximation.

**Results:**

For subject-dependent analysis, the maximum accuracy of 59.17% was obtained using the EEG features that were selected the most (including (i) energy and variance of five frequency bands in frequency-domain feature set, (ii) all feature types in time-domain, time-frequency domain, and nonlinear domain feature sets) and all EEG channels by the Support vector machine algorithm. For subject-independent analysis, the maximum accuracy of 39.30% was obtained using the mostly selected EEG features (which are (i) all feature types excluding the waveform length, average amplitude change value, absolute difference in standard deviation, and slope-change value feature types in time-domain feature set, (ii) the energy and variance values of all frequency bands except gamma frequency band in frequency-domain feature set, (iii) the entropy value of five frequency bands in time-frequency-domain feature set, and (iv) *SD*_2_ and *SD*_1_/*SD*_2_ values where lag = 1 in nonlinear feature set) and EEG channels (which are (i) some definite EEG channels including 2nd, 3rd, 7th, 11th, 13th, 14th, and 15th channels in time-frequency-domain feature set and (ii) all EEG channels in time-domain, frequency-domain, and nonlinear feature sets) by the Support vector machine classifier.

**Discussion:**

Experimental results demonstrate that despite the high-class number, the proposed approach obtained a modest yet considerable advancement in finger movement prediction when the results are compared to the results of similar studies. Additionally, for almost all feature sets, the statistical significance-based feature reduction method improves the prediction performance in the most of classifiers, contributing elaborate EEG channel and feature analysis. Nonetheless, in this study, we used an EEG dataset recorded from only 13 healthy subjects; therefore, a dataset covering more subjects is necessary to reach a more general conclusion.

## 1 Introduction

Brain-computer interface (BCI) systems, which are roughly a hardware and software interaction system, convert brain signals into commands to operate external equipment (Nicolas-Alonso & Gomez-Gil, 2012). The first operational stage of a BCI system is data acquisition to capture brain activities (Schalk et al., 2004). To this purpose, numerous neuroimaging modalities, both invasive and non-invasive, have been used to capture neural activity. Among these modalities, many studies adopted non-invasive ones to reflect brain activity. As one of these non-invasive modalities, electroencephalography (EEG) is widely used as an effective and inexpensive method to reflect brain activity (Chen et al., 2015).

BCI applications can be improved using several control signals (Nicolas-Alonso & Gomez-Gil, 2012). Among these control signals, Motor imagery (MI), an important paradigm of BCI applications, occurs as a thought process throughout which a person imagines a specific action but does not perform it (George et al., 2021). Thus, MI reflects a person's intention of movement to control external devices (Pfurtscheller & Neuper, 2001). To date, in the prediction of MI tasks, large-scale movements of the limbs, including movements of the left hand, right hand, left foot, right foot, both feet and tongue, have been subject of classification (Al-Saegh et al., 2021). However, there exists limited research study that aimed to classify refined MI tasks such as finger movements. Decoding finger movements is considered a difficult task because of the sophistication and refinement of muscle for human finger movements and the volume conduction effect of EEG, which causes a certain degree of aliasing and attenuation (Brunner et al., 2016; Sultana et al., 2023). Moreover, finger movements generate signal amplitudes smaller than those that occur with large-scale movements of the limb (Sultana et al., 2023). To grasp and move objects that are crucial to the daily activities of people, the fingers play an important role (Yang et al., 2024). Therefore, decoding finger movements is vital to provide fine motor control of EEG signals and enhance the daily activities of people with motor disabilities.

EEG signals include temporal, spectral (frequency domain-based and time-frequency domain-based features), spatial, and nonlinear features that can be utilized for the classification of finger movements. In BCI design, the feature extraction step, especially the selection of the feature domain, is one of the profound steps in research because the extracted features directly affect the classification performance (Riaz et al., 2015). To date, many studies have used temporal, spectral, and spatial features to classify finger movements. Common temporal features include mean absolute value, root mean square, standard deviation, waveform length, zero crossing value, variance, and integrated EEG value for finger movement analysis (Kaya et al., 2018; Lee et al., 2021; Mwata-Velu et al., 2022). Several studies have used information collected from frequency domain of EEG signals by evaluating statistical features of power spectrum density of these signals (Kaya et al., 2018). Fourier transform has been mostly investigated to provide frequency-domain-based spectral features. Several Fourier transform-based spectral features such as Fourier transform amplitudes, total power, autoregressive coefficients, power spectral density, and mean power have been investigated to classify finger movements (Kato et al., 2020; Kaya et al., 2018; Khushabe et al., 2011). For spectral feature analysis based on time-frequency domain, the following time-frequency representation methods have been used: Wavelet transform (WT), short-time Fourier transform, and Wavelet packet decomposition (Azizah et al., 2022; Mwata-Velu et al., 2021; Yahya et al., 2019). Spatial domain features have been extensively computed in finger movement classification research (Grosse-Wentrup & Buss, 2008). For spatial domain analysis of EEG activity in finger movement classification, common spatial patterns (CSPs) and their variations such as multiclass common spatial patterns received a great deal of attention (Anam et al., 2020, 2019; Kato et al., 2020), in addition to filter-bank common spatial pattern.

Several feature domains and feature types can be extracted from EEG signals to be utilized for finger movement classification in the literature (Degirmenci et al., 2022, 2023, 2024a,b). However, using irrelevant and redundant features is detrimental to classifier performance (Isler et al., 2015). The irrelevant features increase the computational complexity and cause poor generalization capability for the classifier (Blum & Langley, 1997). Consequently, the selection of a small number of relevant features plays an critical role in classification performance. In this direction, numerous feature reduction methods, including recursive feature reduction (Al Ajrawi et al., 2024), LASSO regression (Huang et al., 2024; Muthukrishnan & Rohini, 2016), correlation-based feature reduction (CFS) (Kabir et al., 2023), maximum relevance minimum redundancy (MRMR) (Kabir et al., 2023), statistical significance-based feature selection (Degirmenci et al., 2024b; Taghizadeh et al., 2021), particle swarm optimization (PSO) (Purushothaman & Vikas, 2018), and genetic algorithm (GA) (Ramos et al., 2016) have been performed as supplementary tools to choose relevant and discriminative features for MI-based BCI system designs.

Numerous classifiers such as decision tree (DT) (Degirmenci et al., 2024a), support vector machines (SVM) (Azizah et al., 2022; Kato et al., 2020; Kaya et al., 2018), k-nearest neighbor (kNN) (Degirmenci et al., 2024a; Wang et al., 2009), Naive Bayes (NB) (Wibawa et al., 2022), Random Forest (RF) (Anam et al., 2019), ensemble learning (EL) (Degirmenci et al., 2024b; Yang et al., 2024) and Neural Networks (NN) (Wang et al., 2009; Wibawa et al., 2022) have been used to classify EEG features for finger movement classification. In addition to machine learning techniques, many studies, which focused on classifying finger movements, presented classification performance of deep learning techniques. Various deep learning techniques were reported to have good classification performance through EEG signal analysis to classify finger movements (Anam et al., 2020; Mwata-Velu et al., 2022; Zahra et al., 2022). Thanks to hidden layers of deep learning structures, complex features can automatically be learned directly from raw EEG signals. Recent studies have concentrated on providing images from EEG signals to use as input data in deep learning models (Alazrai et al., 2019; Mwata-Velu et al., 2022).

Despite the difficulties mentioned above, a few BCI system design studies have been reported in the literature to classify finger movements. There is a need to develop these systems by overcoming these difficulties due to the general structure of finger movements in processing EEG signals through the formulation of new approaches. To date, researchers have concentrated on certain EEG signal analysis methods for finger movement classification. These methods can be listed as follows: (i) some definite temporal features as mentioned above, Fourier transform-based spectral features, and common spatial pattern-based spatial features have been mostly computed through respective methods for the feature extraction step, (ii) several feature selection methods that are complex and are not easy to use have been preferred for feature selection step, and (iii) SVM-based and deep learning-based classification methods have been mostly preferred for classification step. Thus, there is a growing need to analyze different feature domains and different feature types from these domains. Considering the computational load on the BCI systems, it would be better to choose a feature selection method that is effective and easy to use for the feature selection step. From another perspective, in the last decade, deep learning methods, which constantly gain popularity in EEG analysis, have been used to classify finger movements. However, these models have some important drawbacks: (i) deep learning methods need a vast amount of data, (ii) they are not as simple and interpretable as machine learning algorithms, (iii) these systems are identified as “closed black boxes” and are deprivation of transparency, and (iv) training process for deep learning models causes high computational load in real-time BCI system design (Rashid et al., 2020). All these obstacles prevent such systems from being applicable to real life. In addition to SVM-based and deep learning-based approaches, different machine learning algorithms can be investigated for finger movement classification since traditional ML models are simpler and more explicable for humans. Previous studies have utilized a fixed set of EEG channels to extract features for finger movement classification (Azizah et al., 2022; Mwata-Velu et al., 2022). However, existing literature suggests that neural activation patterns are subject-specific and may vary across cerebral hemispheres, even during identical motor imagery (MI) tasks. A comprehensive analysis of feature distributions across EEG channels enables a more precise characterization of underlying neurophysiological processes, thereby underscoring the necessity of identifying electrodes that yield the most informative and discriminative signals for accurate finger movement classification. Recent studies have concentrated on classifying only finger movements, whereas the brain's idle state which is the state that the brain does not perform any mental tasks (NoMT) has been neglected. This situation could lead to an increase in false positives and cause a significant decrease in classification performance (Degirmenci et al., 2024b; Velinchkovsky et al., 1997). Additionally, EEG studies have proved the effectiveness of patterns of brain activity for NoMT state (Collura et al., 1993; Rosazza et al., 2011). The effectiveness of these state networks has been repeated and approved in several neuroimaging and neurophysiological studies (Brookes et al., 2011; Hipp et al., 2012). Therefore, the implementation of the NoMT case as a class may be investigated since it may have an impact on the BCI systems' performance.

This study aims to implement the detailed feature selection and analysis of channel activity in the field of finger movement classification research. Toward this aim, several feature domains, including time domain, frequency domain, time-frequency domain, and nonlinear domain, are used to extract features from raw EEG signals. Four different feature sets are created by extracting different types of features from the relevant feature domains. The effects of these feature sets are investigated separately. The statistical significance-based feature selection method is used to select the most important features. The effect of the feature selection method on each extracted feature set is investigated individually. Feature and channel analysis are performed using statistically significant feature distribution maps, which represent the selected statistically significant feature distribution among 19 EEG channels for each feature set (time domain, frequency domain, time-frequency domain, and nonlinear domain). Several machine learning algorithms are employed for the classification of finger movements. Hence, this study stands out because it pioneers the combined use of detailed features and channel activity analysis. This approach offers a novel perspective in the field of finger movement classification.

### 1.1 Contributions

The following are the main contributions of this study:

- Multiple effective feature domains such as time, frequency, time-frequency, and nonlinear domains were evaluated for the feature extraction process and the effect of these feature sets was analyzed for finger movement classification separately, one by one. In addition, various combinations of these features were provided and investigated for the classification of finger movement.
- The impact of the statistical significance-based feature selection method was analyzed individually for each feature set in relation to the classification of finger movement.
- Feature analysis and channel analysis were conducted by examining the distribution of statistically significant features across 19 EEG channels for both subject-dependent and subject-independent finger movement classifications.
- Various classifier algorithms were comparatively used to evaluate the performance of the sets of extracted features.
- To observe the effect of the brain's resting state, i.e., no mental task (NoMT) case in EEG signals, it was implemented as a class to finger movements' EEG signals in the experimental analysis.

Finally, it is important to highlight that this is the first study to implement, for each feature set, a detailed feature analysis and a channel analysis collectively, alongside investigating the effects of different feature domains on finger movement classification. Additionally, Poincaré plot-based nonlinear features are used for the first time to classify finger movements, to the best of the authors' knowledge.

### 1.2 Organization of the paper

Here is the organization of the remaining sections of this paper: In the “Material and methods” section, details of the dataset in use, feature extraction step, statistically significance-based feature selection process, classification process, and performance evaluation metrics are given. Experimental results of the methods used and discussion of these results are reported in the “Results and Discussion” section. Finally, the main findings of the study are outlined and described in the “Conclusion” section.

## 2 Materials and methods

**Figure 1:**
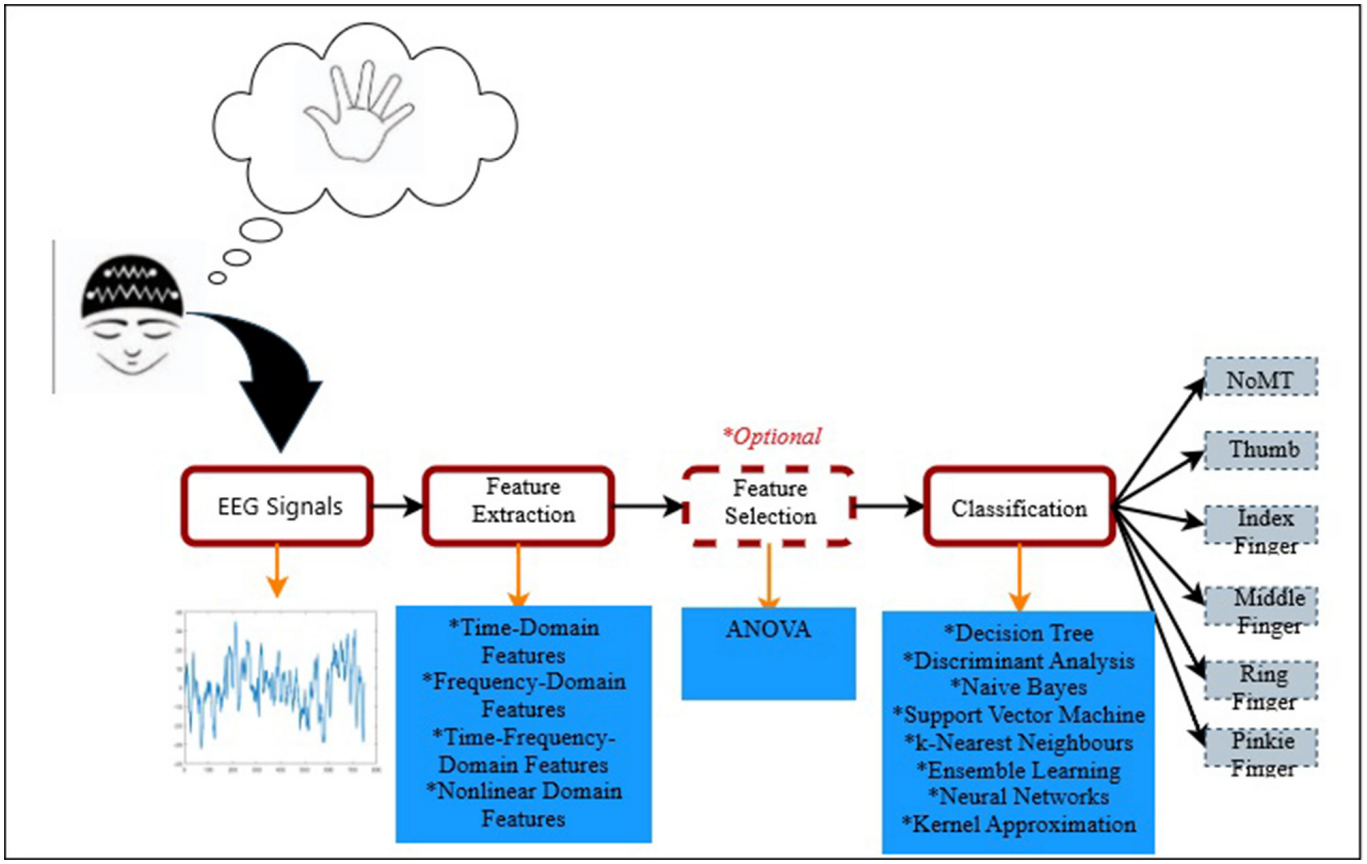
The schematic representation of the proposed methodology for classifying finger movements. EEG signals are segmented into 1-s intervals for the feature extraction process, including time, frequency, time-frequency, and nonlinear domain features. Various well-known classifiers are evaluated to distinguish BCI commands based on the extracted features. The dashed path in the “Feature Selection” block denotes an alternative analysis, where ANOVA is used to select statistically significant features that replace the full set as inputs to the classifiers.

The aim of this study is threefold: to show the advantages of (i) multidirectional EEG analysis which is performed using different feature domains, statistically significance-based feature selection, and various machine learning algorithms, (ii) detailed feature analysis performed using statistically significant feature distribution maps, and (iii) effective channel analysis. Hence, this study was designed and conducted in 4 main sequential stages. These are (i) EEG dataset acquisition, (ii) extraction of features from different domains such as time domain, frequency domain, time-frequency domain, and nonlinear domain, (iii) statistical significance-based feature selection process, (iv) classification, and performance evaluation. The flowchart illustrating the methodology employed in the proposed finger movement classification study is given in Figure 1.

### 2.1 EEG dataset description

In this study, EEG signals are provided from a large electroencephalographic MI dataset for EEG-based BCIs which are recorded and presented by Kaya et al. (2018). This dataset can be found here: [https://figshare.com/collections/A_large_electroencephalographic_motor_imagery_dataset_for_electroencephalographic_brain_computer_interfaces/3917698]. Several types of MIs in 4 different paradigms are available in this dataset. EEG signals were recorded from 13 healthy subjects using the EEG-1200 JE-912A recording system. Using an internationally determined 10–20 electrode placement system, the EEG signals were collected from 19 EEG channels at a sampling frequency of 1,000 Hz. From each subject, EEG signals were recorded for 1 s while the subject implemented the desired MI within this period. This data set includes MI tasks of 10 different body limbs and 4 different BCI paradigms are categorized based on these MI tasks. Among 4 BCI paradigms, Paradigm #3 (5F) and Paradigm #4 (NoMT) are used for the experimental section of this study. In 5F, 5-finger MI tasks including MIs of the five different finger movements on one hand (right or left hand) are available. Participants performed the designated five-finger movement imagery task once during the 1-second presentation of the action signal. Participants imagined flexing the corresponding fingers either upward or downward, according to their individual preference. The marker codes of 5-finger MI tasks are categorized as: (i) Thumb (Class 1), (ii) Index finger (Class 2), (iii) Middle finger (Class 3), (iv) Ring finger (Class 4), and (v) Pinkie (little) finger (Class 5). EEG signals of 5-finger MI tasks from Paradigm #3 (5F) are used in our experimental analysis. In this study, in addition to the 5F paradigm, EEG signals recorded under the NoMT (No MI task) paradigm–characterized by a passive condition in which participants neither engage in motor imagery tasks nor are exposed to visual stimuli–were incorporated into the analysis. Consequently, the dataset comprises six distinct classes of EEG signals, enabling a more comprehensive evaluation of motor-related and non-motor-related neural activity. As the pre-processing step during EEG signal recording, a 0.53–100 Hz band-pass filter is applied. To eliminate the electrical grid interface, a 50 Hz notch filter is applied. After the acquisition of EEG data, 100 samples for each of six different classes were determined for the experimental section to provide a balanced distribution between the classes and to ensure adjusted chance level (Galiotta et al., 2022).

### 2.2 Analysis of EEG signals using various feature extraction domains

To obtain relevant and significant information about EEG signals, the types of EEG features, and the domain of these features are crucial in the feature extraction step. In the literature, spatial features, especially those that are extracted through CSP, have been the most adopted and computed features for finger movement classification. Although, by researchers, spatial features are being utilized the most, we used temporal, spectral, and nonlinear features and investigated their effectiveness for finger movement classification.

#### 2.2.1 Time-domain based feature extraction

At first, using time-domain characteristics of EEG signals, 24 different temporal features were computed separately for all EEG segments. These features present information related to the statistical variations of the EEG signals and their amplitude. Section 3 includes information on the pertinent and distinctive MI time-domain features that we adopted to classify finger movements. “*TDi*” abbreviation represents the time-domain features in this paper.

#### 2.2.2 Frequency-domain based feature extraction

Frequency information embedded in EEG signals was used and spectral features were evaluated during the feature extraction process based on the frequency domain for classification of finger movement. In this direction, the frequency characteristics of the MI EEG signals were obtained on the basis of a fast Fourier Transform. Using these frequency representations, several oscillations that are included within EEG signals such as delta (δ), theta (θ), alpha (α), beta (β), and gamma (γ) waves were obtained. The frequency ranges of these bands is as follows: (i) δ (0.5–4 Hz), (ii) θ (4–8 Hz), (iii), α (8–13 Hz), (iv) β (13–30 Hz), and (v) γ (30–100 Hz). The spectral features were obtained by computing the energy, variance, and spectral entropy values of the extracted frequency bands. These features offer insights into how energy, variance, and irregularity (entropy) vary across the respective frequency bands. These spectral features are evaluated as following Equations 1–3 (Degirmenci et al., 2023; Sayilgan et al., 2021a):


(1) $$Energy_{f}=\sum_{i=1}^{M}y{\left(i\right)}^{2}$$

(2) $$Variance_{f}=\frac{1}{M-1}\cdot \sum_{i=1}^{M}{\left(y_{i}-\bar{y}\right)}^{2}$$

(3) $$Entropy_{f}=\frac{1}{log(M)}\cdot \sum_{i=1}^{M}P(y(i))log(P(y(i))$$

where, f denotes the related frequency band that is used to calculate energy, variance, and spectral entropy values. "M" represents the maximum frequency. “y” denotes the Fourier transform of the related EEG signal and the average value of “y” is denoted as “$\bar{y}$”. The probability that the signal lies within the specified frequency domain is represented as “*P*(*y*(*i*))”.

Section 3 shows the information about the relevant and distinctive MI frequency-domain features that we adopted for the classification of finger movements. ”*FDi”* abbreviation represents the frequency-domain features in this paper.

#### 2.2.3 Time-frequency-domain-based feature extraction

Within the scope of time-frequency-domain feature extraction, the WT is utilized as a time-frequency representation technique to effectively capture and evaluate the underlying time-frequency characteristics of the EEG signals. WT provides both time and frequency information about the EEG signals. Multi-resolution analysis is carried out using its several filters and bandwidths (Sayilgan et al., 2021a). Its working principle is similar to that of a dual Finite-Impulse Response filter (Sayilgan et al., 2021a). EEG signals are separated into high-frequency and low-frequency components using these filters. The identical wavelet coefficients were chosen in both low-pass (LP) and high-pass (HP) filters for the multi-resolution algorithm of WT (Gandhi et al., 2011; Sayilgan et al., 2021a). The scaling parameter, defined by the oscillatory frequency and the length of the wavelet, is connected to the coefficients of the LP filter, while the wavelet function is linked to the coefficients of the HP filter. The outputs of these filters are denoted as the approximation (a) coefficients, and the detail (d) coefficients, respectively. The original EEG time series is entirely decomposed into (a) and (d) coefficients based on the specified decomposition level (Degirmenci et al., 2023).

Wavelet packet decomposition is used to evaluate five distinct EEG sub-bands (δ, θ, α, β, and γ) of each EEG segment. “Haar” mother wavelet and 9-level sub-band decomposition *(i = 1, 2*, ⋯ *, 9)* are applied to extract the Wavelet coefficients of EEG signals sampled at 1000 Hz. According to these parameters, *a*_*i*_ and detail *d*_*i*_ coefficients are decomposed. Subsequently, the relevant coefficient subsets from the decomposition levels are classified according to the frequency domain of EEG sub-bands for the decomposition of EEG signals. The energy, variance, and entropy values of these sub-bands are computed as time-frequency features.

Using the following mathematical formulations (Equations 4 and 5), the energy at each decomposition level is computed as (Gandhi et al., 2011):


(4) $$\begin{array}{l} Energy_{d_{i}}=\overset{N}{\underset{j=1}{\sum }}|d_{ij}{|}^{2},i=1,2,3,\cdots \;,l \end{array}$$

(5) $$\begin{array}{l} Energy_{a_{i}}=\overset{N}{\underset{j=1}{\sum }}|a_{ij}{|}^{2},i=1,2,3,\cdots \;,l \end{array}$$

Here, the detail (*d*_*ij*_) and approximate (*a*_*ij*_) coefficients represent the respective subsets for each frequency band. The wavelet decomposition level, is defined in [1, *l*], is represented as *i = 1,2,3*,⋯ *,l*. "*N*" refers to the number of *d* and *a* coefficients.

The variance value for each decomposition level is computed as follows: Equation 7 (Gandhi et al., 2011):


(6) $$\begin{array}{l} Variance_{i}=\frac{1}{N-1}\cdot \sum_{j=1}^{N}{\left(d_{ij}-\mu_{i}\right)}^{2},i=1,2,3,\cdots ,l\\ \;\mu_{i}=\frac{1}{N}\cdot \sum_{j=1}^{N}d_{ij},i=1,2,3,\cdots ,l \end{array}$$

Here, μ_*i*_ represents the mean of the decomposition level.

The entropy at each decomposition level is calculated as follows Equation 6 (Isler, 2009):


(7) $$\begin{array}{l} Entropy_{i}=\overset{N}{\underset{j=1}{\sum }}d_{ij}^{2}log\left(d_{ij}^{2}\right),i=1,2,3,\cdots \;,l \end{array}$$

Section 3 provides information on the pertinent and distinctive MI time-frequency-domain features that we used for the classification of finger movements. The abbreviation *TF*_*i*_ represents the time-frequency-domain features in this paper.

#### 2.2.4 Nonlinear domain-based feature extraction

In the nonlinear domain feature extraction process, Poincare plot measures are used to capture nonlinear characteristics present in EEG signals. A Poincare plot, a technique derived from nonlinear dynamics, is constructed by plotting each data on the x-axis against subsequent data on the y-axis (Isler, 2009; Cancioglu et al., 2021). In this plot, the overall shape of the distribution is utilized to describe the dynamics of the time series. The Poincaré plot is generated using the raw EEG time series data. An ellipse is adjusted to match the shape of the Poincare plot. The standard deviation of the points' distances in the plot indicates the width (*SD*_1_) and length (*SD*_2_) of the ellipse. Poincare measurements, *SD*_1_ and *SD*_2_, are computed by the following formulas (Equations 8 and 9):


(8) $$\begin{array}{l} SD_{1}=\sqrt{\frac{1}{2}{\left(SDSD\right)}^{2}}=STD\left(\frac{x_{i+lag}-x_{i}}{\sqrt{2}}\right) \end{array}$$

(9) $$\begin{array}{l} SD_{2}=\sqrt{2{\left(SD\right)}^{2}-\frac{1}{2}{\left(SDSD\right)}^{2}}=STD\left(\frac{x_{i+lag}+x_{i}}{\sqrt{2}}\right) \end{array}$$

Here, the standard deviation of successive differences is denoted as *SDSD*, while the standard deviation of the data is represented as *SD* (Brennan et al., 2001; Isler, 2009). Aside from *SD*_1_ and *SD*_2_ values, the product *SD*_1_*SD*_2_ and the ratio $\frac{SD_{1}}{SD_{2}}$ values of these components were also computed. Among the studies in literature that performed Poincare plot measures based on physiological signal analysis, different lag values ranging from 1 to 10 were selected (Contreras et al., 2007). In this study, the four different Poincare plot measures are evaluated using conventional *lag* = 1 condition (Smith et al., 2007).

Section 3 supplies the information about the relevant and distinctive MI nonlinear domain features that we used for finger movement classification. The abbreviation *ND*_*i*_ represents the nonlinear domain features in this paper.

### 2.3 Feature selection using statistical significance (ANOVA)

In the feature selection process, the most discriminative and relevant EEG features were selected using the feature selection method based on statistical significance. Among the statistical significance-based feature selection methods, One-way variance analysis (ANOVA test) was used (Bulut et al., 2022; Degirmenci et al., 2023). The ANOVA test is utilized to compute whether there is a difference between the means in situations involving two or more classes. In total, five (fingers) and one (NoMI) different MI tasks of finger movements were included in our study. Hence, the ANOVA test was preferred to indicate statistically significant features considering the number of classes in this study. The ANOVA test was conducted independently for each of the extracted feature sets, including the time domain, frequency-domain, time-frequency-domain, and nonlinear domain. Performing the ANOVA test allowed us to resolve the statistical significance of the EEG features based on the calculation of the p-values (Narin et al., 2014; Sayilgan et al., 2021b). A statistical significance level (α) of 0.05 was chosen for our analysis (Bulut et al., 2022; Degirmenci et al., 2023; Narin et al., 2014). The features that met the criteria for statistical evidence were identified and designated as statistically significant features. These selected features are fused to create new feature sets with exclusively statistically significant features for each feature domain. As a result, to investigate the efficiency of the recommended feature selection method, two different feature sets were created for each feature domain, including all features and features with statistical significance. Classifications were then performed using these two feature sets in each feature domain.

### 2.4 Classification and performance evaluation

Following the extraction and selection of the different MI EEG features, various machine learning algorithms, including Decision Tree (DT) (Tzallas et al., 2009), Discriminant Analysis (DA) (Hart et al., 2000; Lotte et al., 2018), Naive Bayes (NB) (Hart et al., 2000; Miao et al., 2017), Support Vector Machine (SVM) (Altnkaynak et al., 2020; Chen et al., 2021; Hart et al., 2000), k-Nearest Neighbor (kNN) (Altnkaynak et al., 2020; Hart et al., 2000; Isler, 2009), Ensemble Learning (EL) (Degirmenci et al., 2023; Sayilgan et al., 2019, 2020, 2022), Neural Networks (NN) (Narin & Isler, 2021; Ozdemir et al., 2021; Richard et al., 1991), and Kernel Approximation (KA) (Lei et al., 2019; Maji et al., 2008) were utilized to evaluate the performance of the used methods in relation with classifying five finger movements. The DT separates the data into several subgroups based on its characteristic tree-like structure consisting of branches and nodes, hence, giving the name of the algorithm. In this algorithm, classification is handled based on learning a set of decision rules (Tzallas et al., 2009). As one of the supervised machine learning algorithms, DA aims to accurately separate the independent variables in the feature set into homogeneous groups (Hart et al., 2000; Lotte et al., 2018). NB, as one of the probabilistic classifiers, relies on the Bayes theorem that runs through by examining the membership probability of a sample to all classes in the feature set (Hart et al., 2000; Miao et al., 2017). The main purpose of the SVM algorithm is to achieve the maximum margin between the different data groups to provide a multidimensional hyperplane that best distinguishes between the two or more classes (Altnkaynak et al., 2020; Chen et al., 2021; Hart et al., 2000). kNN is a traditional machine learning algorithm, based on a majority vote among neighbors of a sample to classify it. The highest prevalence of a sample among its k nearest neighbors is examined by this algorithm (Altnkaynak et al., 2020; Hart et al., 2000; Isler, 2009). EL brings together multiple machine learning techniques as a single classifier that provides highly accurate classification (Degirmenci et al., 2023; Sayilgan et al., 2019, 2020, 2022). NNs, in their basic architecture, include three main structures: an input layer, fully connected layers, and an output layer. More advanced NN architectures consist of many layers and each subsequent layer has a connection from the previous layer (Narin & Isler, 2021; Ozdemir et al., 2021; Richard et al., 1991). KA classifier can be used to perform nonlinear classification of data containing many samples (Lei et al., 2019; Maji et al., 2008). A list of the classifiers and corresponding algorithms that were implemented in this study is as follows: (i) Decision Tree: fine, medium, and coarse, (ii) Discriminant Analysis: linear and quadratic, (iii) Naive Bayes: Gaussian and kernel, (iv) Support Vector Machine (SVM): linear, quadratic, cubic, fine Gaussian, medium Gaussian, and coarse Gaussian, (v) k-Nearest Neighbor (kNN): cubic and cosine, (vi) Ensemble Learning: Boosted, Bagged, Subspace Discriminant, Subspace k-NN, and RUSBoosted Trees, (vii) Neural Networks: narrow, medium, wide, bi-layered, and tri-layered, and (viii) Kernel Approximation: support vector machine and logistic regression. Each of these algorithms was adopted using the Classification Learner Toolbox, a component of the Statistics and Machine Learning Toolbox in the Matlab software package version 2023b. As a validation mechanism, to derive a more accurate estimate of the prediction performance of the model, the k-fold cross-validation (CV) method was performed in our simulations. The average classification accuracy is computed after completing k iterations. In our simulations, k is set to 5. In addition, the accuracy performance metric (ACC) is employed to assess the performance of classifiers (Hart et al., 2000). The mathematical expression for the ACC metric is given by Equation 10 (Hart et al., 2000; Isler, 2009):


(10) $$\begin{array}{l} ACC=\frac{TP+TN}{TP+FN+TN+FP} \end{array}$$

Here, the number of correctly predicted EEG segments of finger movements is denoted as TP and TN. On the other hand, the number of EEG segments of finger movements that are incorrectly predicted is denoted as FP and FN.

## 3 Results and discussion

In this study, the classification performance was evaluated for both subject-dependent and subject-independent scenarios across six different feature sets. Feature selection based on statistical significance was applied to enhance model performance. A comparative analysis was conducted using eight machine learning algorithms. Using 19-channel EEG signals, four distinct feature sets were extracted. These feature sets encompass information from four distinct feature domains: time-domain, frequency-domain, time-frequency-domain, and nonlinear domain. In addition to the four primary feature sets, combinations of feature sets derived from different feature domains were also constructed and examined to enhance the classification of finger movements. The impact of each feature set was examined individually. Furthermore, the impact of the ANOVA-based feature selection method was evaluated independently for each feature set. The relevant and discriminative features of all feature sets were selected using ANOVA and the reduced versions of all feature sets were generated to analyze the effectiveness of the ANOVA-based feature selection method. For six different feature sets, all EEG features in their original form, without undergoing any feature selection procedure and the relevant and discriminative EEG features selected from all features were evaluated using different classification algorithms.

The number of all extracted features and ANOVA-selected features for both subject-dependent and subject-independent finger movement classifications is presented in Table 1.

**Table 1:** The sizes of all feature sets, along with the number of features selected using the statistical significance-based feature selection method for finger movement classification, are presented.

**Feature sets**	**Number of all features**	**Number of ANOVA-selected features**								
		**S1**	**S2**	**S3**	**S4**	**S5**	**S6**	**S7**	**S8**	**SI**
TD	456	251	262	377	383	233	264	286	192	313
FD	285	117	98	154	157	119	107	116	67	153
TF	285	10	88	39	136	25	26	135	20	28
ND	76	42	45	53	63	31	33	60	32	38
TD+FD+TF	1,026	378	448	570	676	377	397	537	279	494
TD+FD+TF+ND	1,102	420	493	623	739	408	430	597	311	532

The feature sets under consideration include TD (time-domain feature set), FD (frequency-domain feature set), TF (time-frequency-domain feature set), and ND (nonlinear domain feature set).

All classifiers' performance was tested using both the time-domain features and the ANOVA-selected time-domain features considered in this study, with the results provided in Table 2. In subject-dependent time domain analysis, the SVM algorithm resulted in a maximum of 57.50% accuracy using ANOVA-selected time-domain features which are provided from Subject C (S3). This success within the subject-dependent analysis is also the highest performance result in the time domain. In subject-independent analysis, the highest classification accuracy of 36.20% was yielded using the complete set of time-domain features and SVM algorithm. The impact of applying the ANOVA-based feature selection method to the time-domain features was evaluated, revealing its effect on classification performance. In addition, the spatial distribution of statistically significant time-domain features, identified via ANOVA, across the 19 EEG channels was analyzed. In fact, the channel-wise distribution of features identified as statistically significant and how this distribution influences the classifiers' performance in cases where statistically significant features are used are examined. The distributions of ANOVA-selected, statistically significant time-domain features across the 19 EEG channels for both subject-independent and subject-dependent finger movement classifications are presented in Tables 3 and 4, respectively. In subject-independent analysis, all time domain features, excluding (TD10, TD11, TD12, and TD24), were predominantly identified as statistically significant by the ANOVA test across the majority of EEG channels. The impact of the individual EEG channels was examined, and it was recognized that statistically significant features were chosen from all channels, without any particular focus on specific ones. For subject-independent analysis, this distribution of statistically significant time-domain features did not improved performance in most classifiers. The time-domain features, determined to be statistically significant through a balanced selection process across all feature types and channels for the subject-dependent analysis, are presented in Table 4. This balanced feature distribution improves the classifiers' performance in most of the subjects.

**Table 2:** All classifiers' performances were evaluated in this study using time-domain feature set.

**Components**	**Feature set**	**S1**	**S2**	**S3**	**S4**	**S5**	**S6**	**S7**	**S8**	**SI**
Decision tree	TD	24.20	37.50	38.30	40.00	28.30	35.80	29.20	32.50	28.60
	TD+ANOVA	35.00	38.30	36.00	45.00	24.20	39.20	30.80	31.70	28.50
Discriminant analysis	TD	15.00	26.70	34.20	32.50	20.00	29.20	25.00	26.70	32.10
	TD+ANOVA	34.20	41.70	43.30	46.70	36.70	37.50	20.80	31.70	29.90
Naive Bayes	TD	30.00	40.00	33.30	41.00	26.70	33.30	30.80	38.30	27.90
	TD+ANOVA	25.00	42.50	45.00	48.30	30.00	39.20	30.80	37.50	28.50
Support vector machine	TD	28.30	**50.00**	56.00	48.30	40.00	45.80	28.30	40.80	**36.20**
	TD+ANOVA	35.00	49.20	**57.50**	54.20	39.20	**55.00**	33.30	**41.70**	35.90
k-Nearest Neighbors	TD	**35.80**	44.20	45.00	42.00	34.20	45.00	27.50	38.30	33.50
	TD+ANOVA	29.20	45.00	47.00	50.00	35.00	48.30	30.80	39.20	32.70
Ensemble learning	TD	30.00	44.20	48.30	50.00	38.40	46.70	30.00	37.50	32.60
	TD+ANOVA	33.30	43.30	53.30	**55.80**	**43.30**	50.00	**34.20**	40.80	33.60
Neural networks	TD	29.20	45.00	51.50	47.50	35.80	45.80	31.70	38.30	34.90
	TD+ANOVA	**35.80**	46.70	57.00	50.80	40.80	48.30	**34.20**	40.00	34.70
Kernel approximation	TD	28.33	25.83	34.17	32.50	28.33	24.17	24.17	21.67	25.20
	TD+ANOVA	28.30	27.50	31.00	38.30	23.30	30.00	23.30	30.80	24.70

The maximum accuracy values for each subject-dependent and subject-independent case are highlighted in bold. SI is “Subject-independent”.

**Table 3:** The distribution of statistical significant features, selected via ANOVA, across 19 EEG channels for subject-independent finger movement classification using the time-domain feature set.

	**EEG channels**																			
**Features**	**1**	**2**	**3**	**4**	**5**	**6**	**7**	**8**	**9**	**10**	**11**	**12**	**13**	**14**	**15**	**16**	**17**	**18**	**19**	**Total**
TD1	✓	✓	✓	✓		✓	✓	✓	✓	✓	✓	✓	✓	✓	✓	✓	✓	✓	✓	18
TD2	✓	✓	✓	✓	✓		✓		✓	✓	✓	✓	✓	✓	✓		✓			14
TD3	✓	✓	✓	✓	✓	✓	✓	✓			✓	✓	✓	✓	✓	✓	✓	✓	✓	17
TD4	✓	✓	✓	✓			✓	✓	✓	✓	✓	✓	✓	✓	✓	✓	✓	✓	✓	17
TD5	✓	✓	✓	✓			✓	✓	✓	✓	✓	✓	✓		✓	✓	✓		✓	15
TD6	✓	✓	✓	✓			✓	✓	✓	✓	✓	✓	✓		✓	✓	✓		✓	15
TD7	✓	✓	✓	✓			✓	✓			✓	✓	✓				✓			10
TD8	✓	✓	✓	✓			✓	✓			✓	✓	✓		✓	✓	✓	✓	✓	14
TD9	✓	✓	✓	✓			✓	✓	✓	✓	✓	✓	✓		✓	✓	✓		✓	15
TD10																				0
TD11																				0
TD12															✓					1
TD13	✓	✓		✓			✓	✓	✓	✓	✓	✓		✓			✓		✓	12
TD14	✓	✓	✓	✓	✓	✓	✓	✓	✓	✓	✓	✓	✓	✓	✓	✓	✓	✓	✓	19
TD15	✓	✓	✓	✓			✓	✓			✓	✓	✓		✓	✓	✓	✓	✓	14
TD16	✓	✓	✓	✓			✓	✓	✓	✓	✓	✓	✓		✓	✓	✓	✓	✓	16
TD17	✓	✓	✓	✓			✓	✓	✓	✓	✓	✓	✓	✓		✓	✓	✓	✓	16
TD18	✓	✓	✓	✓			✓	✓	✓	✓	✓	✓	✓	✓	✓	✓	✓	✓	✓	17
TD19	✓	✓	✓	✓	✓	✓	✓	✓	✓	✓	✓	✓	✓	✓	✓	✓	✓	✓	✓	19
TD20	✓	✓	✓	✓	✓	✓	✓	✓			✓	✓	✓	✓		✓	✓	✓	✓	16
TD21	✓	✓	✓	✓	✓	✓	✓	✓			✓	✓	✓	✓			✓			13
TD22	✓	✓	✓	✓	✓	✓	✓	✓		✓	✓	✓	✓	✓		✓	✓	✓	✓	17
TD23	✓	✓	✓	✓	✓	✓	✓	✓	✓	✓	✓	✓		✓	✓	✓	✓		✓	17
TD24														✓						1
Total	20	20	19	20	8	8	20	19	13	14	20	20	18	14	15	16	20	12	17	313

Abbreviations of time-domain features are TD1 (Minumum value), TD2 (Maximum value), TD3 (Mean), TD4 (Standard deviation value), TD5 (Integrated EEG value), TD6 (Mean absolute value), TD7 (Simple square integral), TD8 (Variance), TD9 (Root mean square), TD10 (Waveform length), TD11 (Average amplitude change value), TD12 (Absolute difference in standard deviation), TD13 (Kurtosis), TD14 (Skewness), TD15 (Hjorth parameters (Activity)), TD16 (Hjorth parameters (Mobility)), TD17 (Hjorth parameters (Complexity)), TD18 (Signal range), TD19 (First inter-quartile value (Q1)), TD20 (Second inter-quartile value (Q2)), TD21 (Third inter-quartile value (Q3)), TD22 (Mode value), TD23 (Zero-crossing value), and TD24 (Slope-change value).

**Table 4:** The distribution of statistically significant features, selected via ANOVA, across 19 EEG channels for subject-dependent finger movement classification using the time-domain feature set.

	**EEG channels**																			
**Features**	**1**	**2**	**3**	**4**	**5**	**6**	**7**	**8**	**9**	**10**	**11**	**12**	**13**	**14**	**15**	**16**	**17**	**18**	**19**	**Total**
TD1	7	7	6	7	5	4	5	6	4	6	7	7	6	5	5	5	4	2	5	103
TD2	7	8	7	7	3	5	4	4	4	5	7	6	5	4	3	5	8	2	4	98
TD3	7	6	5	6	7	8	5	6	6	2	7	6	6	6	4	5	8	3	5	108
TD4	8	8	7	8	3	2	4	6	5	7	7	7	3	4	4	6	8	1	4	102
TD5	8	8	6	7	3	3	4	6	5	3	6	6	4	2	4	7	8	2	5	97
TD6	8	8	6	7	3	3	4	6	5	3	6	6	4	2	4	7	8	2	5	97
TD7	8	8	6	7	2	2	2	5	3	4	6	6	4	3	3	3	8	2	3	85
TD8	8	8	7	7	2	2	2	5	3	4	7	6	4	3	3	3	8	2	3	87
TD9	8	8	6	7	3	3	4	6	5	5	6	6	4	3	4	6	8	2	5	99
TD10	2	2	5	4	4	4	3	3	2	3	4	4	6	4	3	4	2	3	3	65
TD11	2	2	5	4	4	4	3	3	2	3	4	4	6	4	3	4	2	3	3	65
TD12	2	3	5	4	4	4	3	3	2	3	4	4	6	3	3	4	2	3	3	65
TD13	6	6	5	6	2	2	2	2	1	2	6	3	5	2	1	2	6	2	6	67
TD14	7	7	6	7	2	3	5	5	5	5	4	7	4	3	5	6	7	1	5	94
TD15	8	8	7	7	2	2	2	5	3	4	7	6	4	3	3	3	8	2	3	87
TD16	7	7	7	7	6	6	7	6	6	7	8	8	5	6	4	5	7	5	8	122
TD17	8	8	6	6	5	6	6	6	6	7	7	7	6	7	5	6	7	4	8	121
TD18	8	8	7	7	2	2	3	5	2	5	7	7	4	4	4	4	8	1	4	92
TD19	6	6	6	7	8	8	4	5	4	3	7	8	6	6	5	3	6	3	4	105
TD20	7	5	5	5	8	8	5	3	6	2	6	6	7	6	6	4	5	3	4	101
TD21	6	7	5	5	8	8	5	2	5	4	7	5	6	6	5	4	7	3	4	102
TD22	6	6	3	7	4	5	5	2	3	3	5	6	3	6	3	4	5	3	3	82
TD23	7	6	6	5	4	4	5	6	6	6	6	5	6	5	4	5	5	3	7	101
TD24	6	6	4	6	5	6	5	5	4	5	4	6	6	7	4	7	5	7	6	104
Total	1-	1-	1-	1-	99	1-	97	1-	97	1-	1-	1-	1-	1-	92	1-	1-	64	1-	2,249
	57	56	38	50		04		11		01	45	42	20	04		12	50		10	

Abbreviations of time-domain features are TD1 (Minumum value), TD2 (Maximum value), TD3 (Mean), TD4 (Standard deviation), TD5 (Integrated EEG value), TD6 (Mean absolute value), TD7 (Simple square integral), TD8 (Variance), TD9 (Root mean square), TD10 (Waveform length), TD11 (Average amplitude change value), TD12 (Absolute difference in standard deviation), TD13 (Kurtosis), TD14 (Skewness), TD15 (Hjorth parameters (Activity)), TD16 (Hjorth parameters (Mobility)), TD17 (Hjorth parameters (Complexity)), TD18 (Signal range), TD19 (First inter-quartile value (Q1)), TD20 (Second inter-quartile value (Q2)), TD21 (Third inter-quartile value (Q3)), TD22 (Mode value), TD23 (Zero-crossing value), and TD24 (Slope-change value).

All classification results obtained using frequency-domain analysis are presented in Table 5. In the subject-dependent analysis, ANOVA-selected frequency-domain features obtained from Subject E (S4) yielded the highest classification accuracy of 55.00% when evaluated using the EL classifier. Within the subject-independent analysis, the SVM classifier achieved the highest accuracy of 30.45% based on frequency-domain features selected through ANOVA. The distribution of statistically significant frequency-domain features, selected via ANOVA, across 19 EEG channels and how this distribution influences classifier performance in cases where statistically significant features are used was investigated. The distribution of frequency-domain features, selected as statistically significant through channel-based ANOVA, across 19 EEG channels for both subject-independent and subject-dependent finger movement classifications is provided in Tables 6 and 7, respectively. For subject-independent analysis, the values of energy and variance across all frequency bands except gamma frequency band are mostly chosen features from frequency-domain feature set as given in Table 6. It is important to note that alpha and beta frequency bands are among the frequency bands where energy and variance values are frequently selected, since the alpha and beta frequency bands are indicated as effective frequency bands providing information about motor activities in literature (Nicolas-Alonso & Gomez-Gil, 2012). However, statistically significant features were distributed in balance among 19 EEG channels. The results indicate that selecting statistically significant features from the frequency-domain feature set contributed to an enhancement in classification performance across nearly all classifiers, except for DA and NN, in the subject-independent classification. For subject-dependent analysis, statistically significant features were intensely chosen, in balance, from all EEG channels, frequency bands, and feature types (especially the values of energy and variance of the frequency bands). When we investigate the performance of feature selection based on ANOVA using the frequency-domain feature set for subject-dependent analysis, the results demonstrate that the superiority of ANOVA-selected frequency domain features over the entire set of frequency-domain features for all subjects as reported in Table 5.

**Table 5:** All classifiers' performances were evaluated in this study using frequency-domain feature set.

**Components**	**Feature set**	**S1**	**S2**	**S3**	**S4**	**S5**	**S6**	**S7**	**S8**	**SI**
Decision tree	FD	29.17	30.83	35.83	35.83	34.17	34.17	34.67	23.33	24.10
2-11	FD+ANOVA	29.17	32.50	33.33	33.33	36.67	25.83	34.17	30.00	25.90
Discriminant analysis	FD	25.00	30.00	40.00	39.17	28.33	35.83	24.17	23.33	28.14
2-11	FD+ANOVA	25.83	35.00	48.33	51.67	41.67	32.50	26.67	31.67	26.60
Naive Bayes	FD	25.00	29.17	35.83	37.50	25.00	36.67	25.00	27.50	23.91
2-11	FD+ANOVA	28.33	30.83	40.83	47.50	31.67	27.50	25.83	24.17	25.00
Support vector machine	FD	28.33	39.17	40.83	40.00	34.17	35.00	29.17	30.83	29.42
2-11	FD+ANOVA	30.00	45.00	50.00	54.17	40.83	40.83	29.17	31.67	**30.45**
k-Nearest Neighbors	FD	26.67	30.83	38.33	36.67	30.83	32.50	29.17	28.33	24.62
2-11	FD+ANOVA	**34.17**	34.17	37.50	45.00	35.83	32.50	29.17	29.17	26.09
Ensemble learning	FD	30.83	38.33	49.17	41.67	40.00	**41.67**	**36.67**	28.33	28.21
2-11	FD+ANOVA	31.67	**45.83**	**51.67**	**55.00**	**47.50**	37.50	33.33	29.17	28.85
Neural networks	FD	28.33	34.17	43.33	44.17	33.33	37.50	29.17	30.00	27.69
2-11	FD+ANOVA	29.17	40.00	50.00	51.67	38.33	38.33	30.00	**35.83**	27.63
Kernel approximation	FD	25.00	20.00	37.50	40.00	24.17	25.83	22.50	33.33	25.71
2-11	FD+ANOVA	26.67	28.33	37.50	40.83	27.50	23.33	25.83	26.67	27.05

The maximum accuracy values for each subject-dependent and subject-independent case are highlighted in bold. SI is “Subject-independent”.

**Table 6:** The distribution of statistical significant features, selected via ANOVA, across 19 EEG channels for subject-independent finger movement classification using the frequency-domain feature set.

	**EEG channels**																			
**Features**	**1**	**2**	**3**	**4**	**5**	**6**	**7**	**8**	**9**	**10**	**11**	**12**	**13**	**14**	**15**	**16**	**17**	**18**	**19**	**Total**
FD1	✓	✓	✓	✓			✓	✓			✓	✓	✓		✓	✓	✓	✓	✓	14
FD2	✓	✓	✓	✓	✓	✓	✓	✓			✓	✓	✓	✓	✓	✓	✓	✓	✓	17
FD3	✓	✓	✓	✓	✓	✓	✓	✓	✓	✓	✓	✓	✓		✓	✓	✓	✓	✓	18
FD4	✓	✓	✓	✓	✓	✓	✓	✓			✓	✓	✓	✓		✓	✓	✓	✓	16
FD5	✓	✓	✓	✓							✓	✓	✓				✓	✓	✓	10
FD6				✓									✓							2
FD7		✓	✓		✓	✓	✓	✓	✓	✓	✓		✓	✓		✓	✓	✓		14
FD8	✓	✓	✓		✓	✓	✓	✓	✓	✓			✓	✓		✓		✓		13
FD9					✓	✓										✓				3
FD10	✓	✓	✓	✓			✓	✓	✓	✓	✓	✓	✓	✓		✓	✓	✓	✓	16
FD11		✓	✓				✓	✓	✓	✓	✓	✓		✓			✓	✓	✓	12
FD12									✓	✓					✓					3
FD13			✓								✓			✓						3
FD14			✓								✓			✓						3
FD15					✓	✓		✓		✓	✓		✓	✓			✓	✓		9
Total	7	9	11	7	7	7	8	9	6	7	11	7	10	9	4	8	9	10	7	153

Abbreviations of frequency-domain features are FD1 (Energy of delta band), FD2 (Variance of delta band), FD3 (Entropy of delta band), FD4 (Energy of theta band), FD5 (Variance of theta band), FD6 (Entropy of theta band), FD7 (Energy of alpha band), FD8 (Variance of alpha band), FD9 (Entropy of alpha band), FD10 (Energy of beta band), FD11 (Variance of beta band), FD12 (Entropy of beta band), FD13 (Energy of gamma band), FD14 (Variance of gamma band), and FD15 (Entropy of gamma band).

**Table 7:** The distribution of statistical significant features, selected via ANOVA, across 19 EEG channels for subject-dependent finger movement classification using the frequency-domain feature set.

	**EEG channels**																			
**Features**	**1**	**2**	**3**	**4**	**5**	**6**	**7**	**8**	**9**	**10**	**11**	**12**	**13**	**14**	**15**	**16**	**17**	**18**	**19**	**Total**
FD1	8	8	8	7	3	3	4	5	4	3	5	8	4	2	5	4	8	3	6	98
FD2	8	8	8	7	3	3	3	5	4	2	4	7	3	3	5	4	8	4	5	94
FD3	6	4	5	4	2	2	4	3	3	2	4	4	2	3	5	3	3	4	4	67
FD4	6	7	3	5	3	2	4	5	3	3	2	6	3	5	5	4	4	7	6	83
FD5	6	6	2	5	0	0	2	3	0	1	1	5	0	4	2	1	3	5	0	46
FD6	2	2	0	2	0	0	0	0	1	1	0	1	1	1	0	0	1	0	1	13
FD7	3	3	1	2	6	5	4	4	2	6	3	4	4	4	4	5	2	4	5	71
FD8	5	5	2	1	5	5	4	4	2	3	2	5	3	4	5	4	2	2	3	66
FD9	1	2	0	0	1	3	2	1	0	0	0	1	1	2	0	2	0	0	0	16
FD10	5	4	6	5	4	4	3	3	3	4	6	6	7	7	2	4	4	3	3	83
FD11	6	5	3	4	3	3	3	3	5	5	5	3	5	6	2	3	3	3	3	73
FD12	4	4	0	1	0	0	2	3	1	2	2	3	1	2	1	3	2	3	2	36
FD13	5	5	5	1	2	2	0	4	2	4	4	5	6	5	2	3	3	1	1	60
FD14	5	5	4	2	2	3	0	4	2	3	4	5	6	5	1	3	6	2	2	64
FD15	3	4	3	4	4	4	4	4	3	3	3	0	0	4	2	3	6	4	5	63
Total	73	72	50	50	38	39	39	51	35	42	45	63	46	57	41	46	55	45	46	933

Abbreviations of frequency-domain features are FD1 (Energy of delta band), FD2 (Variance of delta band), FD3 (Entropy of delta band), FD4 (Energy of theta band), FD5 (Variance of theta band), FD6 (Entropy of theta band), FD7 (Energy of alpha band), FD8 (Variance of alpha band), FD9 (Entropy of alpha band), FD10 (Energy of beta band), FD11 (Variance of beta band), FD12 (Entropy of beta band), FD13 (Energy of gamma band), FD14 (Variance of gamma band), and FD15 (Entropy of gamma band).

Classification results performed using time-frequency-domain features are presented in Table 8. In subject-independent analysis, the highest accuracy of 26.60% was obtained by employing all time-frequency-domain features in conjunction with the EL classifier. In the subject-dependent analysis, the highest classification accuracy 37.50% was achieved for Subject B (S2) using the SVM classifier with the complete set of time-frequency-domain features. It is observed that the classification results provided using time-frequency-domain features are worse than the classification results provided using other feature domains in this study. Tables 9 and 10 present the channel-based distributions of statistically significant features, selected via ANOVA, from the time-frequency-domain feature set for subject-independent and subject-dependent classification, respectively. Based on the experiments conducted, the ANOVA-based feature selection method focused exclusively and predominantly on entropy values across five distinct frequency bands for subject-independent analysis. On the other hand, a quick investigation of channel activity revealed that from some channels, not even a single statistically significant feature was selected. It has been noted that such feature reduction from certain channels and feature types does not increase the classifier performance for subject-independent analysis in any classifier. In subject-dependent analysis, the statistically significant time-frequency-omain features were selected in a comprehensive and balanced manner across all 19 EEG channels and feature types, rather than being focused on specific channels or particular types of features. The effect of this type of statistically significant feature distribution among EEG channels does not improve the classifier performances for subject-dependent analysis in almost all classification models excluding NB, and SVM.

**Table 8:** All classifiers' performances were evaluated in this study using time-frequency-domain feature set.

**Components**	**Feature set**	**S1**	**S2**	**S3**	**S4**	**S5**	**S6**	**S7**	**S8**	**SI**
Decision tree	TF	29.17	30.83	26.67	35.00	22.50	31.67	26.67	20.83	22.44
	TF+ANOVA	20.83	24.17	27.50	33.33	24.17	22.50	29.17	25.00	19.68
Discriminant analysis	TF	17.50	19.17	31.67	30.83	15.83	30.83	17.50	23.33	22.12
	TF+ANOVA	24.17	17.50	30.83	32.50	25.00	30.00	24.17	22.50	20.77
Naive Bayes	TF	29.17	34.17	25.00	31.67	24.17	29.17	24.17	21.67	21.54
	TF+ANOVA	26.67	30.00	31.67	33.33	24.17	31.67	25.00	25.00	19.81
Support vector machine	TF	32.50	**37.50**	30.83	29.17	25.83	30.83	26.67	30.00	22.00
	TF+ANOVA	25.83	31.67	**33.33**	**36.67**	25.83	32.50	27.50	26.67	21.28
k-nearest Neighbors	TF	26.67	34.17	27.50	27.50	24.17	**33.33**	29.17	**30.83**	22.12
	TF+ANOVA	24.17	31.67	**33.33**	32.50	25.00	30.83	28.33	29.17	20.71
Ensemble learning	TF	**35.00**	33.33	28.33	35.83	27.50	31.67	**33.33**	25.83	**26.60**
	TF+ANOVA	25.83	30.83	30.83	**36.67**	25.83	31.67	29.17	24.17	20.77
Neural networks	TF	28.33	30.83	30.83	30.00	18.33	31.67	25.83	25.83	21.22
	TF+ANOVA	22.50	24.17	26.67	31.67	21.67	25.83	23.33	27.50	20.71
Kernel approximation	TF	27.50	28.33	31.67	29.17	**32.50**	29.17	**33.33**	21.67	26.54
	TF+ANOVA	18.33	23.33	26.67	23.33	16.67	31.67	**33.33**	22.50	19.55

The maximum accuracy values for each subject-dependent and subject-independent case are highlighted in bold. SI is “Subject-independent”.

**Table 9:** The distribution of statistical significant features, selected via ANOVA, across 19 EEG channels for subject-independent finger movement classification using the time-frequency-domain feature set.

	**EEG channels**																			
**Features**	**1**	**2**	**3**	**4**	**5**	**6**	**7**	**8**	**9**	**10**	**11**	**12**	**13**	**14**	**15**	**16**	**17**	**18**	**19**	**Total**
TF1																				0
TF2																				0
TF3		✓	✓				✓		✓		✓		✓	✓	✓					8
TF4																				0
TF5																				0
TF6		✓	✓				✓				✓		✓	✓	✓					7
TF7																				0
TF8																				0
TF9		✓	✓				✓						✓	✓	✓					6
TF10																				0
TF11																				0
TF12		✓									✓	✓	✓	✓	✓					6
TF13																				0
TF14																				0
TF15															✓					1
Total	0	4	3	0	0	0	3	0	1	0	3	1	4	4	5	0	0	0	0	28

Abbreviations of time-frequency-domain features are TF1 (Energy of delta band), TF2 (Variance of delta band), TF3 (Entropy of delta band), TF4 (Energy of theta band), TF5 (Variance of theta band), TF6 (Entropy of theta band), TF7 (Energy of alpha band), TF8 (Variance of alpha band), TF9 (Entropy of alpha band), TF10 (Energy of beta band), TF11 (Variance of beta band), TF12 (Entropy of beta band), TF13 (Energy of gamma band), TF14 (Variance of gamma band), and TF15 (Entropy of gamma band).

**Table 10:** The distribution of statistical significant features, selected via ANOVA, across 19 EEG channels for subject-dependent finger movement classification using the time-frequency-domain feature set.

	**EEG channels**																			
**Features**	**1**	**2**	**3**	**4**	**5**	**6**	**7**	**8**	**9**	**10**	**11**	**12**	**13**	**14**	**15**	**16**	**17**	**18**	**19**	**Total**
TF1	3	1	1	1	2	2	1	2	3	3	1	0	4	1	1	2	3	1	3	35
TF2	3	1	1	1	2	2	1	2	3	3	1	0	4	1	1	2	3	1	3	35
TF3	1	5	2	0	2	1	3	1	1	1	2	1	6	3	2	1	3	0	2	37
TF4	3	1	1	1	2	2	1	2	3	3	1	0	4	1	1	2	3	1	3	35
TF5	3	0	1	1	2	2	1	2	3	2	1	0	4	1	1	2	3	1	3	33
TF6	3	4	1	0	3	2	3	2	3	3	2	2	6	4	3	1	4	0	1	47
TF7	3	2	0	1	2	2	0	2	3	3	1	1	4	2	0	2	3	1	2	34
TF8	3	2	0	1	2	2	0	2	3	3	1	1	3	2	0	2	3	1	2	33
TF9	1	5	0	2	0	2	1	4	2	3	1	2	3	4	2	2	4	2	2	42
TF10	3	1	0	1	2	1	0	2	2	2	1	1	4	2	1	3	3	1	2	32
TF11	3	1	0	1	2	1	0	2	2	2	1	1	4	2	0	3	3	1	2	31
TF12	0	0	2	1	1	0	1	0	1	0	1	2	2	2	2	0	3	0	0	18
TF13	3	1	0	2	1	1	0	2	1	1	2	1	3	2	1	2	3	2	2	30
TF14	3	1	0	2	1	1	0	2	1	1	1	1	2	2	1	2	3	2	2	28
TF15	2	1	1	0	0	0	0	0	0	1	0	1	1	1	2	0	1	0	0	11
Total	37	26	10	15	24	21	12	27	31	31	17	14	54	30	18	26	45	14	29	481

Abbreviations of time-frequency-domain features are TF1 (Energy of delta band), TF2 (Variance of delta band), TF3 (Entropy of delta band), TF4 (Energy of theta band), TF5 (Variance of theta band), TF6 (Entropy of theta band), TF7 (Energy of alpha band), TF8 (Variance of alpha band), TF9 (Entropy of alpha band), TF10 (Energy of beta band), TF11 (Variance of beta band), TF12 (Entropy of beta band), TF13 (Energy of gamma band), TF14 (Variance of gamma band), and TF15 (Entropy of gamma band).

The test classification accuracies obtained based on the nonlinear feature set are presented in Table 11. In subject-independent classification, the highest accuracy of 31.76% was obtained using nonlinear features selected via ANOVA in conjunction with the SVM algorithm. Consistent with the subject-independent analysis, the highest classification accuracy of 50.00% in the subject-dependent classification was attained for Subject E (S4) through the application of ANOVA-selected nonlinear features in combination with the SVM classifier. The distributions of statistically significant features selected via ANOVA across the 19 EEG channels for subject-independent and subject-dependent analyses are presented in Tables 12 and 13, respectively. Considering the channel-based statistical significant feature distribution in nonlinear feature set, the role of ANOVA-driven feature selection is explored for nonlinear domain. According to the feature distribution in nonlinear domain for the subject-independent analysis, the features *SD*_2_ and *SD*_1_/*SD*_2_, computed with a lag of 1, were prominently selected through ANOVA. In addition, the statistical significant features were chosen balencedly across all EEG channels with ANOVA. This type selections performed for subject-independent analysis did not improve classification performance in most of the classifiers. Consequently, nonlinear features outperformed the statistical significant nonlinear features in terms of classification accuracy for subject-independent case. The distribution of statistically significant features chosen via ANOVA for subject-dependent analysis is presented in Table 13. The statistical significant features were determined and selected balencedly from all feature types and 19 EEG channels. To demonstrate the impact of nonlinear features, all features were re-examined using the same classifiers, both excluding (TD+FD+TF) and including (TD+FD+TF+ND) nonlinear features. The classifier results are presented in Tables 14 and 15, respectively. Notably, the second combination, which employed the selected features with ANOVA, yielded higher classifier performances. ANOVA-selected nonlinear features provided better classification performance than nonlinear features for subject-dependent analysis in most classifiers (except for DT, SVM, and EL classifiers).

**Table 11:** All classifiers' performances were evaluated in this study using nonlinear domain feature set.

**Components**	**Feature set**	**S1**	**S2**	**S3**	**S4**	**S5**	**S6**	**S7**	**S8**	**SI**
Decision tree	ND	29.17	30.83	29.17	34.17	28.33	34.17	**33.33**	24.17	25.26
	ND+ANOVA	**34.17**	25.83	29.17	41.67	23.33	30.83	**33.33**	28.33	24.42
Discriminant analysis	ND	25.00	33.33	41.67	39.17	30.83	35.00	26.67	34.17	27.24
	ND+ANOVA	30.00	37.50	**45.00**	46.67	32.50	33.33	23.33	30.00	27.05
Naive Bayes	ND	30.83	32.50	30.83	33.33	25.83	31.67	30.00	27.50	23.14
	ND+ANOVA	28.33	33.33	30.83	42.50	29.17	35.00	**33.33**	28.33	21.73
Support vector machine	ND	32.50	**39.17**	40.00	43.33	37.50	35.83	30.00	**35.83**	30.90
	ND+ANOVA	**34.17**	38.33	43.33	**50.00**	35.00	34.17	29.17	33.33	**31.79**
k-Nearest Neighbors	ND	28.33	32.50	36.67	36.67	32.50	35.00	28.33	33.33	30.64
	ND+ANOVA	31.67	35.83	33.33	43.33	30.83	32.50	30.83	28.33	28.27
Ensemble learning	ND	30.83	36.67	44.17	38.33	37.50	35.83	30.00	33.33	29.81
	ND+ANOVA	30.00	36.67	39.17	45.83	**40.83**	35.83	30.83	30.83	27.69
Neural networks	ND	31.67	35.00	**45.00**	35.83	30.83	**36.67**	26.67	30.83	29.36
	ND+ANOVA	30.83	37.50	40.00	42.50	31.67	38.33	30.83	35.00	29.62
Kernel approximation	ND	26.67	28.33	32.50	31.67	24.17	28.33	28.33	24.17	26.09
	ND+ANOVA	24.17	30.00	33.33	38.33	17.50	28.33	29.17	27.50	26.54

The maximum accuracy values for each subject-dependent and subject-independent case are highlighted in bold. SI is “Subject-independent”.

**Table 12:** The distribution of statistical significant features, selected via ANOVA, across 19 EEG channels for subject-independent finger movement classification using the nonlinear domain feature set.

	**EEG channels**																			
**Features**	**1**	**2**	**3**	**4**	**5**	**6**	**7**	**8**	**9**	**10**	**11**	**12**	**13**	**14**	**15**	**16**	**17**	**18**	**19**	**Total**
ND1															✓					1
ND2	✓	✓	✓	✓			✓	✓	✓	✓	✓	✓	✓	✓	✓	✓	✓	✓	✓	17
ND3	✓	✓		✓													✓			4
ND4	✓	✓	✓	✓			✓	✓	✓	✓	✓	✓	✓		✓	✓	✓	✓	✓	16
Total	3	3	2	3	0	0	2	2	2	2	2	2	2	1	3	2	3	2	2	38

Abbreviations of nonlinear domain features are ND1 (*SD*_1_ where lag = 1), ND2 (*SD*_2_ where lag = 1), ND3 (*SD*_1_*SD*_2_ where lag = 1), and ND4 (*SD*_1_/*SD*_2_ where lag = 1).

**Table 13:** The distribution of statistically significant features, selected via ANOVA, across 19 EEG channels for subject-dependent finger movement classification using the nonlinear domain feature set.

	**EEG channels**																			
**Features**	**1**	**2**	**3**	**4**	**5**	**6**	**7**	**8**	**9**	**10**	**11**	**12**	**13**	**14**	**15**	**16**	**17**	**18**	**19**	**Total**
ND1	3	3	5	3	3	3	2	4	3	4	4	3	6	4	3	4	3	2	4	66
ND2	8	8	7	8	1	1	3	5	4	6	6	7	4	4	4	5	8	2	5	96
ND3	6	6	5	5	1	1	3	3	2	4	6	4	4	3	2	2	6	3	4	70
ND4	7	7	6	6	6	6	8	7	6	7	8	8	6	7	5	6	7	6	8	127
Total	24	24	23	22	11	11	16	19	15	21	24	22	20	18	14	17	24	13	21	359

Abbreviations of nonlinear domain features are ND1 (*SD*_1_ where lag = 1), ND2 (*SD*_2_ where lag = 1), ND3 (*SD*_1_*SD*_2_ where lag = 1), and ND4 (*SD*_1_/*SD*_2_ where lag = 1).

**Table 14:** All classifiers' performances were evaluated in this study using the first combination (TD+FD+TF) feature set.

**Components**	**Feature set**	**S1**	**S2**	**S3**	**S4**	**S5**	**S6**	**S7**	**S8**	**SI**
Decision tree	TD+FD+TF	30.00	36.67	35.00	43.33	32.50	33.33	29.17	34.17	31.20
	TD+FD+TF+	29.17	35.00	35.00	44.17	28.33	33.33	25.00	30.00	30.30
	ANOVA									
Discriminant analysis	TD+FD+TF	26.67	28.33	44.17	42.50	27.50	32.50	24.17	24.17	32.40
	TD+FD+TF+	29.17	18.33	25.83	38.33	32.50	35.83	15.00	31.67	34.20
	ANOVA									
Naive Bayes	TD+FD+TF	28.33	33.33	38.33	42.50	29.17	29.17	20.83	32.50	26.20
	TD+FD+TF+	27.50	34.17	36.67	40.83	31.67	31.67	30.00	30.83	27.10
	ANOVA									
Support vector machine	TD+FD+TF	33.33	50.00	**57.50**	51.67	39.17	45.00	28.33	42.50	37.00
	TD+FD+TF+	35.83	**55.83**	55.00	50.00	39.17	48.33	**33.33**	37.50	**38.70**
	ANOVA									
k-Nearest Neighbors	TD+FD+TF	**36.67**	38.33	49.17	40.83	34.17	39.17	31.67	34.17	32.30
	TD+FD+TF+	29.17	45.00	45.00	41.67	35.83	41.67	**33.33**	31.67	32.90
	ANOVA									
Ensemble learning	TD+FD+TF	31.67	41.67	44.17	53.33	33.33	43.33	28.33	35.83	34.70
	TD+FD+TF+	31.67	53.33	51.67	50.83	42.50	46.67	29.17	**45.83**	35.50
	ANOVA									
Neural networks	TD+FD+TF	32.50	44.17	53.33	**55.00**	39.17	**50.00**	32.50	39.17	34.70
	TD+FD+TF+	37.50	54.17	55.83	54.17	**43.33**	47.50	30.83	35.00	36.10
	ANOVA									
Kernel approximation	TD+FD+TF	30.00	24.17	28.33	43.33	28.33	33.33	25.83	25.00	25.40
	TD+FD+TF+	26.67	29.17	26.67	31.67	26.67	25.83	20.00	20.83	26.10
	ANOVA									

The maximum accuracy values for each subject-dependent and subject-independent case are highlighted in bold. SI is “Subject-independent”.

**Table 15:** All classifiers' performances were evaluated in this study using the second combination (TD+FD+TF+ND) feature set.

**Components**	**Feature set**	**S1**	**S2**	**S3**	**S4**	**S5**	**S6**	**S7**	**S8**	**SI**
Decision tree	TD+FD+TF+ND	28.33	33.33	35.00	36.67	36.67	37.50	30.83	35.83	30.30
	TD+FD+TF+	30.00	41.67	36.67	40.00	30.83	36.67	30.83	35.00	30.40
	ND+ANOVA									
Discriminant analysis	TD+FD+TF+ND	25.83	31.67	43.33	38.33	20.00	37.50	24.17	24.17	32.50
	TD+FD+TF+	25.00	15.83	35.83	35.00	36.67	24.17	21.67	31.67	34.40
	ND+ANOVA									
Naive Bayes	TD+FD+TF+ND	26.67	34.17	36.67	35.83	27.50	35.00	31.67	33.33	27.10
	TD+FD+TF+	28.33	39.17	35.00	43.33	31.67	31.67	31.67	35.00	26.90
	ND+ANOVA									
Support vector machine	TD+FD+TF+ND	30.00	**48.33**	55.00	50.00	38.33	42.50	26.67	**41.67**	37.60
	TD+FD+TF+	**36.67**	46.67	**56.67**	**59.17**	41.67	**51.67**	32.50	37.50	**39.30**
	ND+ANOVA									
k-Nearest Neighbors	TD+FD+TF+ND	29.17	43.33	47.50	45.00	30.83	39.17	29.17	32.50	32.10
	TD+FD+TF+	31.67	41.67	43.33	45.00	29.17	45.83	31.67	34.17	33.30
	ND+ANOVA									
Ensemble learning	TD+FD+TF+ND	27.50	40.83	41.67	55.00	36.67	42.50	28.33	40.83	36.20
	TD+FD+TF+	N/A	45.00	50.00	52.50	42.50	44.17	**34.17**	40.83	35.80
	ND+ANOVA									
Neural networks	TD+FD+TF+ND	31.67	42.50	55.83	55.83	35.83	44.17	27.50	40.83	34.40
	TD+FD+TF+	N/A	46.67	55.83	57.50	**45.00**	49.17	30.00	40.83	37.20
	ND+ANOVA									
Kernel approximation	TD+FD+TF+ND	28.33	29.17	28.33	36.67	30.00	23.33	20.83	25.83	26.00
	TD+FD+TF+	N/A	25.00	24.17	38.33	23.33	27.50	22.50	20.83	26.00
	ND+ANOVA									

The maximum accuracy values for each subject-dependent and subject-independent case are highlighted in bold. SI is “Subject-independent”.

**Figure 2:**
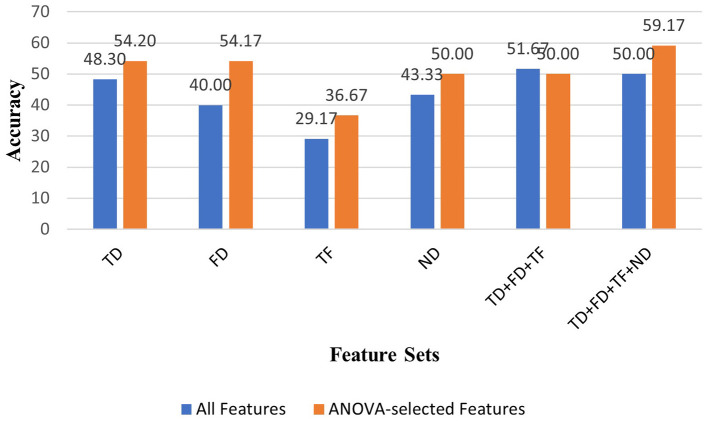
Comparison of classification accuracies obtained using all features vs. ANOVA-selected features across all feature sets with the Support Vector Machine (SVM) classifier for Subject E (S4).

**Figure 3:**
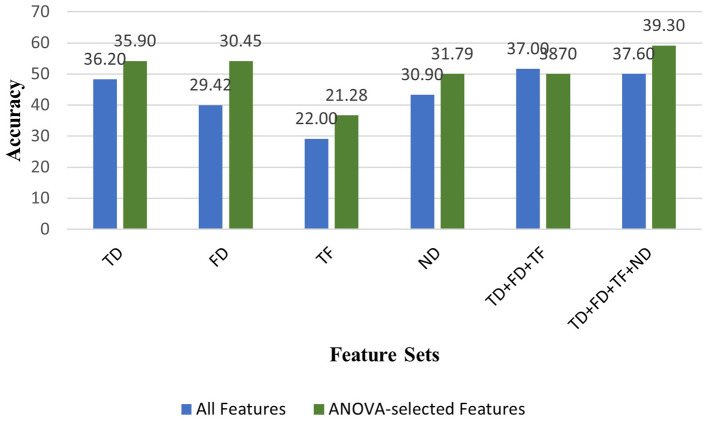
Comparison of classification accuracies obtained using all features vs. ANOVA-selected features across all feature sets with the Support Vector Machine (SVM) classifier in subject-independent analysis.

The primary findings and distinctional aspects of our study on finger movement classification are summarized below:

- We extracted and investigated the different MI EEG features using time-domain, frequency-domain, time-frequency domain, and nonlinear domain of EEG signals.
- This work represents, to the authors' knowledge, the first attempt to apply nonlinear features extracted from Poincare plots in the context of finger movement classification.
- Of all the feature sets analyzed, the second combination (TD+FD+TF+ND), when used with ANOVA, yielded the highest classification performance across both subject-independent and subject-dependent evaluations. Also, the lowest classification performances were mostly provided using Wavelet Transform-based time-frequency features in both cases (Figures 2, 3).
- Several machine learning algorithms were deployed to classify EEG features. Among all algorithms, the highest classification accuracies were generally obtained with SVM algorithm in all feature sets.
- According to experimental results in all feature sets, the ANOVA-based feature selection method mostly improved prediction performance in the majority of machine learning algorithms (Figures 2, 3).
- Contrary to previous studies in the literature that neglect the brain's no mental task condition, we carried out a 6-class classification of finger movements by including the NoMT case instead of excluding the brain's idle state because we aim to propose a more realastic BCI system design. The classification performance of our 6-class finger movement classification study, which we conducted especially for the subject-dependent condition, is superior to the 5-class finger movement classification studies in the literature that eliminate the NoMT condition.
- A comprehensive evaluation of features and EEG channels was performed based on distribution maps highlighting statistically significant features - this approach, proposed for the first time in the context of finger movement classification - is employed to determine efficient and distinctive features and EEG channels.
- Our EEG feature and channel investigation has shown that the ANOVA test selected statistically significant features from some certain EEG frequency bands, and feature types whose effectiveness has been demonstrated in the BCI research field. These feature selections improved the classification performances in some of the analyzed feature sets (especially in frequency domain feature set). Also, in some feature sets (especially in time-domain, time-frequency domain, and nonlinear domain feature sets, used for subject-independent analysis), ANOVA did not prioritize specific EEG channels, frequency bands, and feature types during the feature reduction process. It was concluded that classification performance could be increased with this balanced distribution in some feature sets (especially in nonlinear feature set for subject-dependent analysis). Further analysis of the results revealed that classification performance can improve when using EEG channels and features that have not yet been established as effective in the literature. These findings highlight the critical role of effective feature and channel analysis in enhancing classifier performance.

Beyond these findings, it is important to consider the broader context in which finger movement classification operates, particularly in relation to functional brain networks and neurological disorders. Recent advances in EEG-based BCIs have demonstrated that motor imagery can be effectively used to decode intended movements without any physical action (Pfurtscheller & Neuper, 2001; Wolpaw et. al., 2002). In this context, finger motor imagery classification plays a critical role in enabling control of assistive devices for individuals with motor impairments. By bypassing damaged motor pathways, BCI systems interpret neural activity associated with imagined finger movements and convert it into real-time control commands for devices such as robotic hands or prosthetic fingers (Leeb et al., 2007). This brain-driven control allows voluntary interaction with external systems solely through EEG signals, offering a non-invasive alternative for users with neuromuscular disorders. Therefore, the proposed classification framework not only contributes to improving motor imagery recognition performance but also holds potential as a core component in closed-loop BCI systems aimed at restoring motor function in clinical rehabilitation settings.

We compare the performance of the proposed finger movement classification with that of recent studies that use same EEG dataset with different feature extraction and classification algorithms. The details of these studies are shown in Table 16. In presented studies, BCI systems have proposed with high computational load in terms of feature extraction (Azizah et al., 2022; Yang et al., 2024), and classification (Zahra et al., 2022; Anam et al., 2020; Mwata-Velu et al., 2021, 2022) methods. Despite these computational loads in the system, very high classification performances could not be obtained in both subject-independent (Zahra et al., 2022; Yang et al., 2024) and subject-dependent (Azizah et al., 2022; Mwata-Velu et al., 2021) classifications. In the feature extraction stage, some feature types and categories such as temporal features (Zahra et al., 2022; Alsuradi et al., 2024; Mwata-Velu et al., 2022), Fourier transform amplitudes (Kaya et al., 2018; Yang et al., 2024), and common spatial patterm-based features (Anam et al., 2019, 2020; Kato et al., 2020) have been mainly investigated. Nonlinear features and time-frequency representation methods have been eliminated in this area and their effectiveness should be examined. At the same time, different features should be included in frequently used feature domain categories. In general, SVM (Kaya et al., 2018; Kato et al., 2020; Azizah et al., 2022) and EL (Yang et al., 2024) classifiers have been used to classify finger movements in majority of studies which used machine learning algorithms. Different algorithm approaches can be computed to classify finger movements. On the other hand, literature studies classifying finger movements were only focused on finger movements by eliminating brain's idle case which is the state that the brain is not carrying out any task. NoMT condition can be implemented finger movements because the elimination of the brain's idle case may increase false positive numbers and decrease classication accuracies (Degirmenci et al., 2024b). Among subject-dependent and subject-independent finger movement classification studies performed in literature, the highest classification accuracies have been reported from subject-dependent classifications (Kaya et al., 2018; Alsuradi et al., 2024; Degirmenci et al., 2024b). Compared to the literature studies, different feature domains and feature types (especially Poincare plot-based nonlinear features, entropy-based spectral features, and Wavelet transform-based time-frequency domain features) have been investigated for finger movement classification separately in our study. In addition to SVM and EL algorithms, several machine learning algorithms have used to classify finger movements in our experimental section. Considering feature extraction and classification stages, our study has computational advantages. The EEG signals of NoMT case are implemented to finger movements and a 6-class finger movement classification study to obtain more realistic and accurate BCI system. In finger movement classification research area, the detailed EEG channel and feature investigation have not been performed using different feature domains in any study. Therefore, our main aim is to implement the detailed EEG channel and feature investigation in classification of finger movements using ANOVA-based feature selection method rather than finding the best classifier performance. Toward this aim, ANOVA-based feature selection process conducted to determine discriminative and significant features for each feature set separately. In addition, channel-based ANOVA-selected statistically significant feature distribution maps are obtained for each feature set, separately. Using these maps, the detailed analysis have been performed in this study. In the 5F dataset described by Kaya et al., motor imagery tasks involve finger movements controlled by overlapping regions of the motor cortex, primarily localized around channel C3. Consequently, ERP signals at C3 may not distinctly differentiate between finger movements, with the most notable difference observed between the thumb and pinkie. Additionally, contrary to the common hypothesis in the literature that motor imagery predominantly focuses on channels C3, C4, and Cz (Pfurtscheller & Neuper, 2001), our findings reveal a more widespread distribution of neural activity across multiple EEG channels within specific feature domains. This suggests that brain regions beyond the primary motor cortex contribute meaningfully to finger movement representation (Pfurtscheller & Neuper, 2006). Previous studies have utilized a fixed set of EEG channels to extract features for finger movement classification (Azizah et al., 2022; Mwata-Velu et al., 2022). However, existing literature suggests that neural activation patterns are subject-specific and may vary across cerebral hemispheres, even during identical MI tasks. A comprehensive analysis of feature distributions across EEG channels enables a more precise characterization of underlying neurophysiological processes, thereby underscoring the necessity of identifying electrodes that yield the most informative and discriminative signals for accurate finger movement classification. Given the essential role of functional brain networks in motor planning and execution (Deco et al., 2011), such broad EEG analyses offer valuable insights into overall brain dynamics. Additionally, distinguishing finger movements from resting states (NoMT) holds significant potential for clinical applications, including the diagnosis and rehabilitation of neurological disorders such as Parkinson's disease and stroke-induced motor deficits (Ward et al., 2005). Future integration of functional brain network data with advanced classification models may enhance early detection and facilitate personalized treatment strategies for nervous system disorders. This aspect of the study has brought an important perspective to the studies on the classification of finger movements.

**Table 16:** A comparison of the accuracy of this method with several state-of-the-art studies, for both subject-independent and subject-dependent cases, within this field.

**Study**	***N***	**n**	**Features**	**Classifier**	**c**	**CV**	**Accuracy (%)**
**Subject-independent task**							
Kaya et al. (2018)	8	19	PSD	SVM	5	random split	43.00
			EEG band power			(63-27-10%)	
			FT amplitudes				
Zahra et al. (2022)	8	19	EEG time series	CNN	5	10-fold	57.50
			Sliding window				
			Noise addition				
Yang et al. (2024)	8	19	Feature-dependent	EL	5	5-fold	50.64
			frequency band				
			selection				
			FT amplitudes				
			Riemanian geometry				
Alsuradi et al. (2024)	8	19	Temporal features	N/A	5	leave-one-	40.00
						subject-out	
Degirmenci et al. (2024b)	8	19	ITD	SVM	6	5-fold	34.48
**This study**	**8**	**19**	**TD, FD, TF**,	**SVM**	**6**	**5-fold**	**39.30**
			**and ND features**				
**Subject-dependent task**							
Kaya et al. (2018)	8	19	PSD	SVM	5	random split	20.00-60.00
			EEG band power			(63-27-10%)	
			FT amplitudes				
Anam et al. (2019)	4	19	CSP	RF	5	5-fold	51.00-56.00
Anam et al. (2020)	4	19	CSP	ADL	5	5-fold	74.61-77.75
Kato et al. (2020)	8	19	multi-class CSP	SVM	5	10-fold	23.90-58.30
Mwata-Velu et al. (2021)	8	4	EMD	BLS	5	200-fold	66.00-76.13
Azizah et al. (2022)	8	4	Spectrogram features	SVM	5	10-fold	21.20-66.60
Mwata-Velu et al. (2022)	4	4	Raw EEG data	EEGNet	5	200-fold	80.10-91.70
Alsuradi et al. (2024)	8	19	Temporal features	N/A	5	N/A	Average 50.00
							within-subject
Degirmenci et al. (2024b)	8	19	ITD	EL	6	5-fold	35.83-55.00
**This study**	**8**	**19**	**TD, FD, TF**,	**SVM**	**6**	**5-fold**	**32.50-59.17**
			**and ND features**				

The abbreviation “N” represents the “number of subjects,” “n” denotes the “number of EEG channels,” “c” refers to the “number of classes,” and “CV” stands for the “Cross-Validation Method”. The classifiers used are CNN (Convolutional Neural Network), RF (Random Forest), ADL (Autonomous Deep Learning), SVM (Support Vector Machine), EEGNet (EEGNet Deep Learning Model), BLS (Bi-layered Long-Short Classifier), and EL (Ensemble Learning). Features are PSD (power spectral density), FT (Fourier transfrom), ITD (intrinsic time-scale decomposition), EMD (empirical mode decomposition), and CSP (common spatial pattern).

## 4 Conclusion

In this study, the effects of different feature domains and a statistical significance-based feature selection method are investigated to classify finger movements. First, several EEG features of EEG segments are obtained from four different feature domains, including time-domain, frequency-domain, time-frequency, and nonlinear domain. In addition to these feature sets, two different combinations of features from multiple domains were investigated. Hence, a total of 1102 EEG features are calculated from four different feature domains, and six feature sets are generated for our finger movement analysis. By applying the statistical significance-based feature selection method, relevant and significant – hence fewer – EEG features are determined and selected from each feature set separately. All features obtained in six different feature sets and the statistically significant reduced feature sets were tested with various machine learning algorithms. The applied methods were tested on two different classification cases (subject-dependent and subject-independent classification). The results showed that the highest accuracy rates of 39.30% and 59.17% were obtained using the second combination feature set (TD + FD + TF + ND) and the SVM classifier in subject-independent and subject-dependent classifications, respectively. The selected EEG features (which are (i) energy and variance of five frequency bands in frequency-domain feature set, (ii) all feature types in time domain, time-frequency-domain, and nonlinear domain feature sets) and all EEG channels resulted in maximum accuracy of 59.17% with the SVM classifier for subject-dependent analysis.

For subject-independent analysis, the selected EEG features included (i) all feature types except waveform length, average amplitude change value, absolute difference in standard deviation, and slope-change value in the time-domain feature set; (ii) energy and variance values of all frequency bands except the gamma band in the frequency-domain feature set; (iii) entropy values of five frequency bands in the time-frequency-domain feature set; and (iv) *SD*_2_ and *SD*_1_/*SD*_2_ values with lag = 1 in the nonlinear feature set. The selected EEG channels comprised (i) specific channels including the 2nd, 3rd, 7th, 11th, 13th, 14th, and 15th channels in the time-frequency domain feature set, and (ii) all EEG channels in the time-domain, frequency-domain, and nonlinear feature sets. This combination achieved a maximum accuracy of 39.30% with the SVM classifier. According to the detailed feature and channel activity research, it was concluded that the selected features and channels may vary depending on whether the classification case is subject-dependent or subject-independent. The ANOVA-based feature selection method generally improves the prediction performance in choosing significant and relevant EEG features from different feature sets. The best classification results on two different classification cases were obtained with the second combination feature set (TD+FD+TF+ND) among all feature sets and the SVM classifier among all classification algorithms.

Our main goal in this study is not to provide the best classifier performance but to investigate and indicate the discriminative EEG channels and features for the classification of finger movements. Toward this aim, the EEG channel and feature analysis was performed using channel-based statistical feature distribution maps. Particularly, the detailed feature and EEG channel analysis was implemented for the first time to classify finger movements along with various feature domains and their different combinations to the best of our knowledge. The EEG channel and feature analysis in finger movement classification indicates that it works well and supports literature in choosing specific frequency bands, and features from some feature domains. These specific selections have also been demonstrated to be effective in previous studies of prediction of motor imagery tasks based on EEG. It has been observed that selections are performed from features and channels that are not proven in the literature and that this improves the classification performance. Conversely, performance was also improved by balanced selections across all channels and features. Therefore, our experimental analysis suggests that classifier performance may be improved by implementing detailed feature and EEG channel analysis using a feature selection method based on statistical significance. Nonetheless, in this study, we only used an EEG dataset recorded from 13 healthy subjects, and each EEG data consists of 19 EEG channels sampled at 1000 Hz. To draw a more general conclusion, a dataset covering more subjects is necessary.

## Data Availability

Publicly available datasets were analyzed in this study. This data can be found here: https://www.nature.com/articles/sdata2018211.

## References

[B1] Al Ajrawi S. Rao R. Sarkar M. (2024). A hierarchical recursive feature elimination algorithm to develop brain computer interface application of user behavior for statistical reasoning and decision making. J. Neurosci. Methods 408:110161. 10.1016/j.jneumeth.2024.110161 38718901

[B2] Alazrai R. Abuhijleh M. Alwanni H. Daoud M. I. (2019). A deep learning framework for decoding motor imagery tasks of the same hand using EEG signals. IEEE Access 7, 109612–109627. 10.1109/ACCESS.2019.2934018

[B3] Al-Saegh A. Dawwd S. A. Abdul-Jabbar J. M. (2021). Deep learning for motor imagery EEG-based classification: a review. Biomed. Signal Process. Control 63:102172. 10.1016/j.bspc.2020.102172

[B4] Alsuradi H. Khattak A. Fakhry A. Eid M. (2024). Individual-finger motor imagery classification: a data-driven approach with Shapley-informed augmentation. J. Neural Eng. 21:026013. 10.1088/1741-2552/ad33b3 38479013

[B5] Altinkaynak M. Dolu N. Guven A. Pektas F. Ozmen S. Demirci E.. (2020). Diagnosis of attention deficit hyperactivity disorder with combined time and frequency features. Biocybernet. Biomed. Eng. 40, 927–937. 10.1016/j.bbe.2020.04.006

[B6] Anam K. Bukhori S. Hanggara F. S. Pratama M. (2020). “Subject-independent classification on brain-computer interface using autonomous deep learning for finger movement recognition,” in 42nd Annual International Conference of the IEEE Engineering in Medicine & *Biology Society (EMBC)* (Montreal, QC: IEEE), 447–450. 10.1109/EMBC44109.2020.9175718 33018024

[B7] Anam K. Nuh M. Al-Jumaily A. (2019). “Comparison of EEG pattern recognition of motor imagery for finger movement classification,” in 6th International Conference on Electrical Engineering, Computer Science and Informatics (EECSI) (EECSI: Bandung), 24–27. 10.23919/EECSI48112.2019.8977037

[B8] Azizah R. N. Zakaria H. Hermanto B. R. (2022). Channels selection for pattern recognition of five fingers motor imagery electroencephalography signals. J. Phys. 2312:012019. 10.1088/1742-6596/2312/1/012019

[B9] Blum A. L. Langley P. (1997). Selection of relevant features and examples in machine learning. Artif. Intell. 97, 245–271. 10.1016/S0004-3702(97)00063-5

[B10] Brennan M. Palaniswami M. Kamen P. (2001). Do existing measures of poincare plot geometry reflect nonlinear features of heart rate variability? IEEE Trans. Biomed. Eng. 48, 1342–1347. 10.1109/10.959330 11686633

[B11] Brookes M. J. Woolrich M. Luckhoo H. Price D. Hale J. R. Stephenson M. C.. (2011). Investigating the electrophysiological basis of resting state networks using magnetoencephalography. Proc. Nat. Acad. Sci. 108, 16783–16788. 10.1073/pnas.1112685108 21930901 PMC3189080

[B12] Brunner C. Billinger M. Seeber M. Mullen T. R. Makeig S. (2016). Volume conduction influences scalp-based connectivity estimates. Front. Comput. Neurosci. 10:121. 10.3389/fncom.2016.00121 27920674 PMC5119053

[B13] Bulut A. Ozturk G. Kaya I. (2022). Classification of sleep stages via machine learning algorithms. J. Intellig. Syst. Appl. 5, 66–70. 10.54856/jiswa.202205210

[B14] Cancioglu E. Sahin S. Isler Y. (2021). Poincare Çizimi Ölçümlerinden Topluluk Öğrenmesi Yöntemleri Kullanılarak Proses Kontrol Sistemlerinde Arıza Tespit ve Teşhisi. Avrupa Bilim ve Teknoloji Dergisi. 26, 30–34. 10.31590/ejosat.952761

[B15] Chen T. Antoniou G. Adamou M. Tachmazidis I. Su P. (2021). Automatic diagnosis of attention deficit hyperactivity disorder using machine learning. Appl. Artif. Intellig. 35, 657–669. 10.1080/08839514.2021.1933761

[B16] Chen X. Wang Y. Nakanishi M. Gao X. Jung T. P. Gao S. (2015). High-speed spelling with a noninvasive brain-computer interface. Proc. Nat. Acad. Sci. 112, E6058–E6067. 10.1073/pnas.1508080112 26483479 PMC4640776

[B17] Collura T. F. (1993). History and evolution of electroencephalographic instruments and techniques. J. Clini. Neurophysiol. 10, 476–504. 10.1097/00004691-199310000-00007 8308144

[B18] Contreras P. Canetti R. Migliaro E. R. (2007). Correlations between frequencydomain HRV indices and lagged Poincare plot width in healthy and diabetic subjects. Physiol. Meas. 28, 85–94. 10.1088/0967-3334/28/1/008 17151422

[B19] Deco G. Jirsa V. K. McIntosh A. R. (2011). Emerging concepts for the dynamical organization of resting-state activity in the brain. Nat. Rev. Neurosci. 12, 43–56. 10.1038/nrn2961 21170073

[B20] Degirmenci M. Yuce Y. K. Isler Y. (2022). “Motor imaginary task classification using statistically significant time-domain EEG features,” in 2022 30th Signal Processing and Communications Applications Conference (SIU) (Safranbolu: IEEE), 1–4. 10.1109/SIU55565.2022.9864745

[B21] Degirmenci M. Yuce Y. K. Isler Y. (2024a). İstatistiksel anlamlı zaman alanı EEG özniteliklerinden el parmak hareketlerinin sınıflandırılması. *Gazi Universitesi Mühendislik Mimarl*&#x00131;k Fakültesi Dergisi 39, 1597–1610. 10.17341/gazimmfd.1241334

[B22] Degirmenci M. Yuce Y. K. Perc M. Isler Y. (2023). Statistically significant features improve binary and multiple motor imagery tasks predictions from EEGs. Front. Hum. Neurosci. 17:1223307. 10.3389/fnhum.2023.1223307 37497042 PMC10366537

[B23] Degirmenci M. Yuce Y. K. Perc M. Isler Y. (2024b). EEG-based finger movement classification with intrinsic time-scale decomposition. Front. Hum. Neurosci. 18:1362135. 10.3389/fnhum.2024.1362135 38505099 PMC10948500

[B24] Galiotta V. Quattrociocchi I. D'Ippolito M. Schettini F. Aricò P. Sdoia S.. (2022). EEG-based brain-computer interfaces for people with disorders of consciousness: features and applications. A systematic review. Front. Hum. Neurosci. 16:1040816. 10.3389/fnhum.2022.1040816 36545350 PMC9760911

[B25] Gandhi T. Panigrahi K. B. Anand S. (2011). A comparative study of wavelet families for EEG signal classification. Neurocomputing 74, 3051–3057. 10.1016/j.neucom.2011.04.029

[B26] George O. Smith R. Madiraju P. Yahyasoltani N. Ahamed S. I. (2021). "Motor Imagery: A review of existing techniques, challenges and potentials," in *2021 IEEE 45th Annual Computers, Software, and Applications Conference (COMPSAC)* (Madrid: IEEE), 1893–1899.

[B27] Grosse-Wentrup M. Buss M. (2008). Multiclass common spatial patterns and information theoretic feature extraction. IEEE Trans. Biomed. Eng. 55, 1991–2000. 10.1109/TBME.2008.921154 18632362

[B28] Hart P. E. Stork D. G. Duda R. O. (2000). Pattern Classification (2nd ed.). Hoboken, NJ: A Wiley-Interscience Publication.

[B29] Hipp J. F. Hawellek D. J. Corbetta M. Siegel M. Engel A. K. (2012). Large-scale cortical correlation structure of spontaneous oscillatory activity. Nat. Neurosci. 15, 884–890. 10.1038/nn.3101 22561454 PMC3861400

[B30] Huang W. Liu X. Yang W. Li Y. Sun Q. Kong X. (2024). Motor imagery EEG signal classification using distinctive feature fusion with adaptive structural LASSO. Sensors 24:3755. 10.3390/s24123755 38931540 PMC11207242

[B31] Isler Y. (2009). A Detailed Analysis of the Effects of Various Combinations of Heart Rate Variability Indices in Congestive Heart Failure (Ph.D. thesis). Dokuz Eylul University, Izmir, Turkey.

[B32] Isler Y. Narin A. Ozer M. (2015). Comparison of the effects of cross-validation methods on determining performances of classifiers used in diagnosing congestive heart failure. Measurem. Sci. Rev. 15, 196–201. 10.1515/msr-2015-0027

[B33] Kabir M. H. Mahmood S. Al Shiam A. Musa Miah A. S. Shin J. Molla M. K. I. (2023). Investigating feature selection techniques to enhance the performance of EEG-based motor imagery tasks classification. Mathematics 11:1921. 10.3390/math1108192117946502

[B34] Kato M. Kanoga S. Hoshino T. Fukami T. (2020). “Motor imagery classification of finger motions using multiclass CSP,” in 2020 42nd Annual International Conference of the IEEE Engineering in Medicine & *Biology Society (EMBC)* (Montreal, QC: IEEE), 2991–2994. 10.1109/EMBC44109.2020.9176612 33018634

[B35] Kaya M. Binli M. K. Ozbay E. Yanar H. Mishchenko Y. (2018). A large electroencephalographic motor imagery dataset for electroencephalographic brain computer interfaces. Scientific Data 5, 1–16. 10.1038/sdata.2018.211 30325349 PMC6190745

[B36] Khushaba R. N. Kodagoda S. Liu D. Dissanayake G. (2011). “Electromyogram (EMG) based fingers movement recognition using neighborhood preserving analysis with QR-decomposition,” in *2011 Seventh International Conference on Intelligent Sensors, Sensor Networks and Information Processing* (Adelaide, SA: Sensor Networks and Information), 1–105.

[B37] Lee K. H. Min J. Y. Byun S. (2021). Electromyogram-based classification of hand and finger gestures using artificial neural networks. Sensors 22:225. 10.3390/s22010225 35009768 PMC8749583

[B38] Leeb R. Lee F. Keinrath C. Scherer R. Bischof H. Pfurtscheller G. (2007). Brain-computer communication: motivation, aim, and impact of exploring a virtual apartment. IEEE Trans. Neural Syst. Rehabilit. Eng. 15, 473–482. 10.1109/TNSRE.2007.906956 18198704

[B39] Lei D. Tang J. Li Z. Wu Y. (2019). Using low-rank approximations to speed up kernel logistic regression algorithm. IEEE Access 7, 84242–84252. 10.1109/ACCESS.2019.2924542

[B40] Lotte F. Baugrain L. Cichocki A. Clerc M. Congedo M. Rakotomamonjy A.. (2018). A review of classification algorithms for EEG-based brain-computer interfaces: a 10 year update. J. Neural Eng. 15:031005. 10.1088/1741-2552/aab2f2 29488902

[B41] Maji S. Berg A. C. Malik J. (2008). “Classification using intersection kernel support vector machines is efficient,” in 2008 IEEE Conference on Computer Vision and Pattern Recognition (Anchorage, AK: IEEE), 1–8.

[B42] Miao M. Zeng H. Wang A. Zhao C. Liu F. (2017). Discriminative spatial-frequency-temporal feature extraction and classification of motor imagery EEG: an sparse regression and Weighted Naïve Bayesian Classifier-based approach. J. Neurosci. Methods 278, 13–24. 10.1016/j.jneumeth.2016.12.010 28012854

[B43] Muthukrishnan R. Rohini R. (2016). “LASSO: A feature selection technique in predictive modeling for machine learning,” in 2016 IEEE International Conference on Advances in Computer Applications (ICACA) (Coimbatore, India: IEEE), 18–20. 10.1109/ICACA.2016.7887916

[B44] Mwata-Velu T. Y. Avina-Cervantes J. G. Cruz-Duarte J. M. Rostro-Gonzalez H. Ruiz-Pinales J. (2021). Imaginary finger movements decoding using empirical mode decomposition and a stacked BiLSTM architecture. Mathematics 9:3297. 10.3390/math9243297

[B45] Mwata-Velu T. Y. Avina-Cervantes J. G. Ruiz-Pinales J. Garcia-Calva T. A. González-Barbosa E. A. Hurtado-Ramos J. B.. (2022). Improving motor imagery EEG classification based on channel selection using a deep learning architecture. Mathematics 10:2302. 10.3390/math10132302

[B46] Narin A. Isler Y. (2021). Detection of new coronavirus disease from chest x-ray images using pre-trained convolutional neural networks. J. Faculty of Eng. Architect. Gazi Univer. 36, 2095–2107. 10.17341/gazimmfd.827921

[B47] Narin A. Isler Y. Ozer M. (2014). Investigating the performance improvement of HRV Indices in CHF using feature selection methods based on backward elimination and statistical significance. Comp. Biol. Med. 45, 72–79. 10.1016/j.compbiomed.2013.11.016 24480166

[B48] Nicolas-Alonso L. F. Gomez-Gil J. (2012). Brain computer interfaces, a review. Sensors 12, 1211–1279. 10.3390/s120201211 22438708 PMC3304110

[B49] Ozdemir M. A. Degirmenci M. Izci E. Akan A. (2021). EEG-based emotion recognition with deep convolutional neural networks. Biomed. Eng. 66, 43–57. 10.1515/bmt-2019-0306 32845859

[B50] Pfurtscheller G. Neuper C. (2001). Motor imagery and direct brain-computer communication. Proc. IEEE 89, 1123–1134. 10.1109/5.939829

[B51] Pfurtscheller G. Neuper C. (2006). Future prospects of ERD/ERS in the context of brain-computer interface (BCI) developments. Prog. Brain Res. 159, 433–437. 10.1016/S0079-6123(06)59028-4 17071247

[B52] Purushothaman G. Vikas R. (2018). Identification of a feature selection based pattern recognition scheme for finger movement recognition from multichannel EMG signals. Austral. Phys. Eng. Sci. Med. 41, 549–559. 10.1007/s13246-018-0646-7 29744809

[B53] Ramos A. C. Hernández R. G. Vellasco M. (2016). “Feature selection methods applied to motor imagery task classification,” in 2016 IEEE Latin American Conference on Computational Intelligence (LA-CCI) (Cartagena: IEEE), 1–6.

[B54] Rashid M. Sulaiman N. Majeed A. A. P. P. Musa R. M. Ab Nasir A. F. Bari B. S.. (2020). Current status, challenges, and possible solutions of EEG-based brain-computer interface: a comprehensive review. Front. Neurorobot. 14:25. 10.3389/fnbot.2020.00025 32581758 PMC7283463

[B55] Riaz F. Hassan A. Rehman S. Niazi I. K. Dremstrup K. (2015). EMD-based temporal and spectral features for the classification of EEG signals using supervised learning. IEEE Trans. Neural Syst. Rehabilit. Eng. 24, 28–35. 10.1109/TNSRE.2015.2441835 26068546

[B56] Richard M. D. Lippmann R. P. (1991). Neural network classifiers estimate Bayesian a posteriori probabilities. Neural Comput. 3, 461–483. 10.1162/neco.1991.3.4.461 31167331

[B57] Rosazza C. Minati L. (2011). Resting-state brain networks: literature review and clinical applications. Neurol. Sci. 32, 773–785. 10.1007/s10072-011-0636-y 21667095

[B58] Sayilgan E. Yuce Y. K. Isler Y. (2019). Prediction of evoking frequency from steady-state visual evoked frequency. Natural Eng. Sci. 4, 91–99.

[B59] Sayilgan E. Yuce Y. K. Isler Y. (2020). Determining gaze information from steady-state visually-evoked potentials. Karaelmas Fen ve Mühendislik Dergisi 10, 151–157. 10.7212/zkufbd.v10i2.158840512634

[B60] Sayilgan E. Yuce Y. K. Isler Y. (2021a). “Evaluating steady-state visually evoked potentials-based brain-computer interface system using wavelet features and various machine learning methods,” in Brain-Computer Interface (London: IntechOpen). 10.5772/intechopen.98335

[B61] Sayilgan E. Yuce Y. K. Isler Y. (2021b). Evaluation of wavelet features selected via statistical evidence from steady-state visually-evoked potentials to predict the stimulating frequency. J. Faculty Eng. Architect.Gazi Univers. 36, 593–605. 10.17341/gazimmfd.664583

[B62] Sayilgan E. Yuce Y. K. Isler Y. (2022). Investigating the effect of flickering frequency pair and mother wavelet selection in steady-state visually-evoked potentials on two-command brain-computer interfaces. IRBM 43, 594–603. 10.1016/j.irbm.2022.04.006

[B63] Schalk G. McFarland D. J. Hinterberger T. Birbaumer N. Wolpaw J. R. (2004). BCI2000: a general-purpose brain-computer interface (BCI) system. IEEE Trans. Biomed. Eng. 51, 1034–1043. 10.1109/TBME.2004.827072 15188875

[B64] Smith A. L. Reynolds K. J. Owen H. (2007). Correlated Poincare indices for measuring heart rate variability. Austral. Phys. Eng. Sci. Med. 30, 336–341. 18274076

[B65] Sultana A. Ahmed F. Alam M. S. (2023). A systematic review on surface electromyography-based classification system for identifying hand and finger movements. Healthcare Analyt. 3:100126. 10.1016/j.health.2022.100126

[B66] Taghizadeh Z. Rashidi S. Shalbaf A. (2021). Finger movements classification based on fractional fourier transform coefficients extracted from surface emg signals. Biomed. Signal Process. Control 68:102573. 10.1016/j.bspc.2021.102573

[B67] Tzallas A. T. Tsipouras M. G. Fotiadis D. I. (2009). Epileptic seizure detection in EEGs using time-frequency analysis. IEEE Trans. Inform. Technol. Biomed. 13, 703–710. 10.1109/TITB.2009.2017939 19304486

[B68] Velichkovsky B. Sprenger A. Unema P. (1997). “Towards gaze-mediated interaction: collecting solutions of the “Midas touch problem”, in *Human-Computer Interaction INTERACT'97, IFIP-The International Federation for Information Processing* (Boston, MA: Springer).

[B69] Wang B. Wong C. M. Wan F. Mak P. U. Mak P. I. Vai M. I. (2009). “Comparison of different classification methods for EEG-based brain computer interfaces: a case study,” in 2009 International Conference on Information and Automation (Zhuhai/Macau: ICIA), 1416–1421.

[B70] Ward N. S. (2005). Neural plasticity and recovery of function. Prog. Brain Res. 150, 527–535. 10.1016/S0079-6123(05)50036-0 16186046

[B71] Wibawa A. D. Mohammad B. S. Y. Fata M. A. K. Nuraini F. A. Prasetyo A. Pamungkas Y. (2022). “Comparison of EEG-based biometrics system using naive bayes, neural network, and support vector machine,” in 2022 International Conference on Electrical and Information Technology (IEIT)(Malang: IEIT), 408–413.

[B72] Wolpaw J. R. Birbaumer N. McFarlanda D. J. Pfurtscheller V. T. M. (2002). Brain-computer interfaces for communication and control. Clini. Neurophysiol. 113, 767–791. 10.1016/S1388-2457(02)00057-3 12048038

[B73] Yahya N. Musa H. Ong Z. Y. Elamvazuthi I. (2019). Classification of motor functions from electroencephalogram (EEG) signals based on an integrated method comprised of common spatial pattern and wavelet transform framework. Sensors 19:4878. 10.3390/s19224878 31717412 PMC6891287

[B74] Yang K. Li R. Xu J. Zhu L. Kong W. Zhang J. (2024). DSFE: Decoding EEG-based finger motor imagery using feature-dependent frequency, feature fusion and ensemble learning. IEEE J. Biomed. Health Inform. 28, 4625–4635. 10.1109/JBHI.2024.3395910 38709613

[B75] Zahra H. N. Zakaria H. Hermanto B. R. (2022). Exploration of pattern recognition methods for motor imagery EEG signal with convolutional neural network approach. J. Phys. 2312:012064. 10.1088/1742-6596/2312/1/012064

